# Factor VIII Is Synthesized in Human Endothelial Cells, Packaged in Weibel-Palade Bodies and Secreted Bound to ULVWF Strings

**DOI:** 10.1371/journal.pone.0140740

**Published:** 2015-10-16

**Authors:** Nancy A. Turner, Joel L. Moake

**Affiliations:** Department of Bioengineering, Rice University, Houston, Texas, United States of America; Emory University School of Medicine, UNITED STATES

## Abstract

The cellular synthesis site and ensuing storage location for human factor VIII (FVIII), the coagulation protein deficient in hemophilia A, has been elusive. FVIII stability and half-life is dependent on non-covalent complex formation with von Willebrand factor (VWF) to avoid proteolysis and clearance. VWF is synthesized in megakaryocytes and endothelial cells, and is stored and secreted from platelet alpha granules and Weibel-Palade bodies of endothelial cells. In this paper we provide direct evidence for FVIII synthesis in 2 types of primary human endothelial cells: glomerular microvascular endothelial cells (GMVECs) and umbilical vein endothelial cells (HUVECs). Gene expression quantified by real time PCR revealed that levels of *F8* and *VWF* are similar in GMVECs and HUVECs. Previous clinical studies have shown that stimulation of vasopressin V2 receptors causes parallel secretion of both proteins. In this study, we found that both endothelial cell types express *AVPR2* (vasopressin V2 receptor gene) and that *AVPR2* mRNA levels are 5-fold higher in GMVECs than HUVECs. FVIII and VWF proteins were detected by fluorescent microscopy in Weibel-Palade bodies within GMVECs and HUVECs using antibodies proven to be target specific. Visual presence of FVIII and VWF in Weibel-Palade bodies was confirmed by correlation measurements. The high extent of correlation was compared with negative correlation values obtained from FVIII detection with cytoplasmic proteins, β-actin and Factor H. FVIII activity was positive in GMVEC and HUVEC cell lysates. Stimulated GMVECs and HUVECs were found to secrete cell-anchored ultra-large VWF strings covered with bound FVIII.

## Introduction

Human factor VIII (FVIII) functions as a cofactor for activated factor IX, and mutations in the FVIII gene (*F8*) result in hemophilia A. [1] FVIII in plasma is proteolyzed and/or pinocytosed unless it is protected by non-covalent binding to soluble forms of von Willebrand factor (VWF) multimers. [2–4]

VWF is synthesized in megakaryocytes and endothelial cells (ECs), and is stored in megakaryocyte/platelet alpha granules and EC Weibel-Palade bodies (WPBs). [5] VWF multimers are secreted from WPBs in ultra-long, hyper-adhesive strings anchored to EC surfaces. Platelets adhere to these secreted/anchored strings and initiate hemostasis. [6,7]

Although the synthesis of FVIII has been demonstrated in many human tissue types, including liver, spleen, kidney and lymphatic tissue, [8–10] ECs are the only specific human cell type where direct evidence of FVIII production has been obtained. FVIII protein or activity levels, either released into culture medium or from cell lysates, have been measured in human liver sinusoidal (LS) ECs, cardiac microvascular (MV) ECs, intestinal MVECs, dermal MVECs, lung MVECs and pulmonary artery ECs. [11–14] Positive *F8* mRNA expression has been reported for each of these EC types, except intestinal MVECs. [12–14] However, because of the variety of ECs, and even after extensive investigation, the precise nature of FVIII synthesis, storage and secretion in ECs remains unclear.

Clinical data showing parallel increases in plasma FVIII and VWF levels in mild hemophilia A patients and Type 1 VWD patients following des-amino-D-arginine vasopressin (DDAVP) administration has lead to speculation that FVIII, in addition to VWF, is stored within human ECs. [15–17] Experimental proof of FVIII secretion with DDAVP stimulation has not been reported for unaltered (non-transfected) human ECs (or any other natural non-EC cell type).

Transcripts for *F8* have been found in murine hepatocytes and LSECs, and cultured murine LSECs release measurable amounts FVIII activity. [18] Recently, two articles reported data confirming murine ECs as the exclusive cellular source of FVIII synthesis in mice. [19,20] These two articles are compatible with the previous human data reported by Shahani, et al., who used receptor-specific cell sorting to isolate LSECs from hepatocytes in liver tissues to show that LSECs are the primary source of FVIII synthesis within the liver. [12]

In this report, we demonstrate FVIII synthesis in two types of unmodified, primary human ECs: glomerular microvascular endothelial cells (GMVECs) and umbilical vein endothelial cells (HUVECs) for the first time. The FVIII synthesis was confirmed by *F8* gene expression, immunofluorescent microscopic FVIII protein detection, and measurements of FVIII activity. We also show that FVIII is stored in WPBs along with VWF, in both EC types; and that stimulated GMVECs and HUVECs secrete cell-anchored ultra-large (UL) VWF strings with FVIII bound to them.

## Methods

### Ethics Statement

The Rice Institutional Review Board (IRB) approved of the human tissue collection and the experiments on human endothelial cells in this study. The unidentified tissue samples (designated for disposal) were collected under a protocol approved by the Rice IRB. The Rice IRB waived the need for consent. Protocol Name: Processing of Large von Willebrand Factor (VWF) Multimers: VWF Cleavage, Thrombosis and Platelet Aggregation, Protocol Number: 11-183E. The Rice IRB reviews protocols annually and has approved of this consent procedure and study through 5/13/2016.

### Human Tissue Culture

#### Human glomerular microvascular endothelial cells (GMVECs)

Primary GMVECs at passage 2 were purchased from Cell Systems (ACBRI 128, Kirkland, WA) and grown to confluence in MCDB basal medium (M131, Sigma-Aldrich) with additions of microvascular growth supplement (MVGS, Life Technologies) and penicillin, streptomycin and glutamine (PSQ, Life Technologies). Cell type was verified by presence of VWF in WPBs by fluorescent microscopy. GMVECs were used in experiments at passages 3–6 and not stained earlier than 11 days post seeding. All 3 human cell types used in this study were removed from tissue culture flasks non-enzymatically by incubation with 5 mM EDTA in Ca^+2^, Mg^+2^-free PBS and gentle cell scraping.

#### Human umbilical vein endothelial cells (HUVECs)

Primary HUVECs were detached and then pooled from 5–8 human umbilical veins per isolation. Umbilical cords were washed with phosphate buffer (140 mM NaCl, 0.4 mM KCl, 1.3 mM NaH_2_PO_4_, 1.0 mM Na_2_HPO_4_, 0.2% glucose, pH 7.4) and then infused with a collagenase solution (0.02% in PBS) for 30 min at room temperature. The cords were rinsed with 100 ml of phosphate buffer, and solutions containing endothelial cells were centrifuged at 250g for 10 min. HUVECs were maintained in M131 with additions of low-serum growth supplement (LSGS, Life Technologies) and PSQ. Cell type was confirmed by presence of VWF in WPBs by fluorescent microscopy. HUVECs were used at passages 2–7 in these studies, and not stained earlier than 6 days post-seeding.

#### Human adult dermal fibroblasts (Fibroblasts)

Fibroblasts were purchased from American Type Culture Collection (PCS-201-012, Manassas, VA) and maintained in M131 plus PSQ and 10% fetal bovine serum (Atlanta Biologicals). The fibroblasts were studied in parallel with the endothelial cells as negative controls. Cell type was confirmed by the presence of Fibroblast Surface Protein by fluorescent microscopy. Fibroblasts in these experiments were used at passages 4–6, and cells were not stained earlier than 4 days post-seeding.

### Relative Quantitative Gene Expression

The gene expression levels of *F8* (Entrez Gene ID 2157), *VWF* (ID 7450), *AVPR2* (ID 554) and *GAPDH* (ID 2597) were measured in GMVECs (11–23 days post-seeding), HUVECs (7–14 days post-seeding), and fibroblasts (6–7 days post-seeding). Prior to RNA extraction, cells were maintained for 24 hours in serum-free medium consisting of M131 with PSQ plus insulin, transferrin and selenium (ITS, Life Technologies). RNA was isolated using TRIzol (Life Technologies), chloroform extraction and isopropanol precipitation. RNA integrity was verified by 260/280 optical density ratios and 1%-agarose-formaldehyde electrophoresis, and was reverse transcribed using SuperScript III Supermix (Life Technologies). Samples (100 ng cDNA) were amplified in triplicate by real-time polymerase chain reaction (PCR) with the conditions: 95°C for 3 min, 40 cycles of (10 sec at 95°C, 10 sec at 55°C, 30 sec at 72°C), and 95°C for 10 sec followed by melting curves from 65° to 95°C (CFX96, BioRad). Amplified products were detected using TaqMan Gene Expression Assays (S1 Table) (with 6-carboxyfluorescein-labeled probes that span target exon junctions) and Fast Advanced Master Mix (Life Technologies, Carlsbad, CA).

#### Relative quantification measurements

The relative quantification of gene expression levels were calculated as described in the Applied Biosystems User Bulletin No. 2 (P/N 4303859) and by Livak and Schmittgen. [21]

The mRNA copy numbers in each cell type were calculated as follows:

Gene mRNA copy numbers = 2^–CT^, where C_T_ is the average threshold cycle number of the gene of interest (S3 Table). The normalized gene mRNA copy number is the copy number of the gene of interest divided by the copy number for GAPDH (S4 Table).

The quantification of each gene in each cell type relative to the expression in GMVECs was calculated using the following expressions:

The change in threshold cycle for each gene (in each cell type) = ΔC_T_:

$$\Delta {\text{C}}_{\text{T}}=\;{\text{C}}_{\text{T}}\left(\text{gene}\right)\;-\;{\text{C}}_{\text{T}}\left(\text{GAPDH}\right).$$

The change in threshold cycle number relative to GMVECs = ΔΔC_T_:

$$\Delta \Delta {\text{C}}_{\text{T GMVECs}}=\;\Delta {\text{C}}_{\text{T}}\left(\text{gene in GMVECs}\right)\;-\;\Delta {\text{C}}_{\text{T}}\left(\text{gene in GMVECs}\right)$$

$$\Delta \Delta {\text{C}}_{\text{T HUVECs}}=\;\Delta {\text{C}}_{\text{T}}\left(\text{gene in HUVECs}\right)\;-\;\Delta {\text{C}}_{\text{T}}\left(\text{gene in GMVECs}\right)$$

$$\Delta \Delta {\text{C}}_{\text{T Fibroblasts}}=\;\Delta {\text{C}}_{\text{T}}\left(\text{gene in Fibroblasts}\right)\;–\;\Delta {\text{C}}_{\text{T}}\left(\text{gene in GMVECs}\right)$$

The fold-changes relative to GMVECs are calculated by raising 2 to the negative power of the ΔΔC_T_ value for each gene. For each GMVEC gene ΔΔC_T_ = 0, and 2^0^ = 1.

Standard Deviation (SD) Calculations

$$\text{S}1\;=\text{ Average SD of }{\text{C}}_{\text{T}}\text{ for each gene}$$

$$\text{S}2\;=\text{ Average SD of }{\text{C}}_{\text{T}}\text{ for GAPDH}$$

$$\text{SD for }\Delta \Delta {\text{C}}_{\text{T}}\;=\text{ square root }({\text{S}}_{1}{}^{2}+\;{\text{S}}_{2}{}^{2})$$

The ranges for the relative amounts are calculated by evaluating 2^-ΔΔCT^ plus the SD (low range value) and 2^-ΔΔCT^ minus the SD (high range value).

### FVIII and VWF Immunoblots

#### Recombinant (r) FVIII standard

1 vial of Helixate FS (Bayer Healthcare) containing 252 IU was diluted with 2 ml water (final concentration = 0.126 IU/μl containing 12.6 ng/μl of rFVIII protein). For gel separation, 22 μl of 12.6 ng/μl rFVIII was diluted with 10 μl of SDS-containing sample buffer (BioRad) and heated at 95°C for 5 min. 15 μl of this mixture was loaded per gel lane containing ~130 ng of rFVIII.

#### Plasma purified VWF standard

Plasma VWF was purified from 5 units of cryoprecipitate obtained from the Gulf Coast Regional Blood Center (Houston, TX). The VWF was isolated by glycine and NaCl precipitation followed by size-exclusion fractionation. [22] The VWF concentrations were determined by fluorescent immunoassay. Gels were loaded with 15 μl samples containing 90–160 ng of reduced, denatured VWF.

Samples were electrophoresed into 4–15% polyacrylamide gels (BioRad), stained with Bio-Safe Coomassie G-250 and transferred to PVDF membranes. Membranes were incubated separately and detected for FVIII using mouse monoclonal anti-human FVIII (Thermo Fisher Scientific; MA1-10589) plus goat anti-mouse-HRP (Rockland Immunochemicals, cat. #RL610-1302) and detected for VWF with either rabbit anti-human VWF plus donkey anti-rabbit-HRP (Life Technologies, cat. #A16029) or with goat anti-human VWF (Bethyl Laboratories, cat. #A80-138) plus donkey anti-goat-HRP (Life Technologies, cat. #A15999). Each blot was incubated with StrepTactin-HRP conjugate (for molecular weight detection) and chemiluminescent reagents (WesternC, BioRad), before digital imaging (ChemiDoc XRS, BioRad).

### Immunofluorescent Microscopy

#### Microscope instrumentation

Fluorescent images were acquired using IP Lab software version 3.9.4r4 with a fluorescence colocalization module (Scanalytics, Inc., Fairfax, VA) on a Nikon Diaphot TE300 microscope equipped with CFI Plan Fluor 60× oil, numerical aperture 1.4 and CFI Plan Apo Lambda 100× oil, numerical aperture 1.45 objectives plus 10× projection lens (Nikon, Garden City, NY), SensiCamQE CCD camera (Cooke Corp., Romulus, MI), motorized stage and dual filter wheels (Prior) with single band excitation and emission filters for FITC/TRITC/CY5/DAPI (Chroma, Rockingham, VT). The image size acquired at 60× is 78 μm × 58 μm and the image size acquired at 100× is 41 μm × 30 μm. Calibration bars on images are 10 μm.

#### Fluorescent secondary detection antibodies

The Alexa Fluor (AF)-labeled secondary antibodies were used at final concentrations of 20 μg/ml and were purchased from Life Technologies (S2 Table).

#### Internal cell staining

Cells grown on gelatin-coated glass coverslips (1-mm thick) were washed with PBS, fixed with 1% p-formaldehyde in PBS, and then treated with 0.02% Triton-X to allow internal staining. The cells were stained for 15 min with the relevant primary and fluorescent antibody pairs. The cell nuclei were detected with 1.5 μg/ml 4’,6-diamidino-2-phenylindole (DAPI) included in the mounting medium (Fluoro-Gel II, Electron Microscopy Sciences). In experiments where only cell surfaces were detected with antibodies, the treatment with Triton-X was omitted.

#### Fluorescent emission “cross-talk” controls

Two separate coverslips of HUVECs were treated and stained for internal detection of VWF in WPBs, each with a separate secondary fluorescently labeled antibody. HUVECs on coverslip 1 were stained with rabbit anti-VWF plus chicken anti-rabbit IgG AF-488 (green) (cat. #A21441) and the HUVECs on coverslip 2 were stained with rabbit anti-VWF plus chicken anti-rabbit IgG AF-647 (red) (cat. #A21443). Both coverslips were imaged at 488 nm and at 647 nm and merged with DAPI images.

### Internal Detection of FVIII and VWF

The following primary and fluorescently labeled secondary antibody pairs were used to detect internal FVIII concurrently with VWF in both GMVECs and HUVECs:

#### FVIII and VWF detection antibodies

10 μg/ml mouse monoclonal anti-human FVIII antibody clone F8-5.5.72 (Thermo Fisher Scientific; MA1-10589) plus secondary antibody goat anti-mouse F(ab’)_2_ fragment-IgG AF-647 (cat. #A21237); and 10 μg/ml polyclonal rabbit anti-human VWF (Ramco Laboratories, Sugarland, TX) plus secondary antibody chicken anti-rabbit IgG AF-488 (cat. #A21441). The monoclonal antibody against FVIII detects full length human FVIII and does not cross-react with VWF. The antigen used to generate the polyclonal rabbit anti-human VWF antibody was VWF purified in our laboratory from human cryoprecipitate purchased at the Gulf Coast Regional Blood Center (Houston, TX).

#### Fibroblast surface staining

Fibroblast cell surfaces were stained with Fibroblast Surface Protein (FSP) separately or in conjunction with internal cell staining to confirm cell type. For surface staining, the fibroblasts were washed, fixed and then stained with 10 μg/ml mouse monoclonal against human FSP (Abcam, clone 1B10, ab11333) plus goat anti-mouse IgM AF-488 (cat. #A20142). In surface staining followed by internal staining, the cells were fixed again (to retain antibodies to surface proteins) before treatment with Triton-X and then stained with detection antibody pairs.

### Internal Detection of FVIII with β-actin or Factor H

The following primary and fluorescently labeled secondary antibody pairs were used to detect internal FVIII concurrently either with β-actin or with Factor H in HUVECs:

#### FVIII and β-actin detection antibodies

10 μg/ml mouse monoclonal anti-human FVIII antibody clone F8-5.5.72 plus secondary donkey anti-mouse IgG AF-488 (cat. #A21202); and 8 μg/ml polyclonal goat anti-β -actin (I-19, Santa Cruz Biotechnology, sc-1616) plus secondary antibody chicken anti-goat IgG AF-647.

#### FVIII and Factor H detection antibodies

10 μg/ml mouse monoclonal anti-human FVIII antibody clone F8-5.5.72 plus secondary antibody donkey anti-mouse IgG AF-488 (cat. # A21202); and 10 μg/ml polyclonal goat anti-human Factor H (Complement Technologies, Tyler, TX; cat. #A237) plus secondary antibody chicken anti-goat IgG AF-647 (cat. #A21469). The polyclonal antibody made against human Factor H has previously been shown to be monospecific in detecting Factor H protein in human serum and in Factor H-depleted serum by Western blot techniques. [23]

### Fluorescence Colocalization Measurements

FVIII locations and detection intensities within GMVECs and HUVECs were compared with internal detection of VWF. In HUVECs, internal detection of FVIII was additionally compared with internal detection of β-actin and Factor H. FVIII was compared with these proteins for similarities in shape and location by measuring Pearson’s correlation coefficient (PCC) and by graphs of intensity values at specific locations on merged images. Prior to analysis, the images at 60× and 100× had non-subjective background subtraction.

#### Pearson’s correlation coefficient measurements and scatter plots

The extent of similarity between two channels was determined using intensity scatter plots and software calculated values of Pearson’s correlation coefficient (PCC). [24–26] PCC is a statistical measure of strength of correlation that ranges from -1 to 1, where negative values show an inverse relationship between the two probes; zero indicates no correlation; and 1 indicates complete correlation between the two channels. The PCC provides shape correlation by comparing the intensity distribution of the two channels. PCC values are independent of high background levels. In most cases, scatter plots of green channel intensity (y-axis) verses red channel intensity (x-axis) from a merged image of 2 spatially correlated proteins should show a single linear relationship between the two channels. A merged image with perfect overlap distribution of the red and green channels at equal intensities will result in a centered line (where y = x) and a Pearson’s coefficient value of 1. Values for PCC between 0.5 and 1 indicate a degree of correlation in analysis of biological immunofluorescent images. [26]

#### Graphs of intensity measurements in WPBs

Intensities from the 488-nm and 647-nm channels were measured along a 600-pixel distance in merged images of internal FVIII staining in WPBs concurrently with VWF (in GMVECs and HUVECs), and with β-actin or with Factor H (in HUVECs). The intensity graphs are “slices” of intensity values from each channel along this line in merged fluorescent images. A single intensity measurement was made per pixel (1 pixel = 0.11 μm at 60X and 1 pixel = 0.058 μm at 100X) along the 600-pixel long line. Plots of the green intensity values and the red intensity values at the same locations allowed a visual demonstration of signal colocalization. The graphs are intended to be visual aids showing (for FVIII and VWF) that the red and green intensities change in value in a synchronized manner at each image location. Measurements were made with images obtained at 60× and 100× where the 600-pixel length corresponds to 68 μm and 35 μm, respectively.

### FVIII detection on EC-secreted/anchored ULVWF strings

#### GMVEC and HUVEC stimulation

GMVECs and HUVECs on coverslips were washed with PBS and stimulated with 100μM histamine in PBS (± 2.5 mM EGTA/MgCl_2_ to minimize ADAMTS-13 cleavage) [22] for 2 min. Cells were then stained with rabbit anti-VWF plus chicken anti-rabbit IgG AF-488 for 15 min, washed with PBS and fixed. Following fixation, the cells were washed with PBS and stained with mouse monoclonal anti-human FVIII plus goat anti-mouse F(ab’)_2_ fragment-IgG AF-647. In these experiments, GMVECs (at passage 4 and 6) were stimulated and stained from 8 to 10 days after seeding. HUVECs (at passage 2 and 4) were stimulated and stained from 6 to 7 days after seeding.

#### FVIII and VWF intensity measurements on ULVWF strings

Intensities from the 488-nm (VWF, green) and 647-nm (FVIII, red) channels were measured at each pixel (1 pixel = 0.11 μm at 60X and 1 pixel = 0.058 μm at 100X) along the ULVWF strings from merged images of VWF and FVIII detection in GMVECs and HUVECs after histamine stimulation. The intensity graphs are the intensity values from each channel measured at locations traced along the VWF strings in the green-channel fluorescent image. The lengths of the ULVWF strings (converted to microns) and the ratios of FVIII intensity to VWF intensity for each string were calculated in each cell type from images obtained at 60× and 100×. Graphs were generated of the red and green intensities versus the distance along ULVWF strings (in pixels) to demonstrate the process used to obtain the intensity data. In images at 60×, 100 pixels = 11.4 μm and in images at 100×, 200 pixels = 11.8 μm. The intensity graphs of measurements made along VWF strings with shorter lengths appear to be lower in resolution.

### GMVEC and HUVEC samples for FVIII activity measurements

#### GMVEC and HUVEC lysates

GMVECs and HUVECs in T-75 flasks were washed with PBS and incubated with 0.5 ml of mammalian cell lysis reagent (CelLytic M, Sigma-Aldrich, C2978) for 15 min at room temperature with rocking. Cell lysates were collected using a cell scraper, centrifuged at 10,000g for 10 min at 4°C, and supernatants were immediately frozen at -80°C in pre-chilled tubes until assayed. GMVECs lysates were at passages 4 and 6; HUVECs lysates were at passages 1, 2, 5, 6 and 7.

#### FVIII activity assay

FVIII activity was measured in EC lysates using a modified version of the Chromogenix Coatest SP4 FVIII Activity Assay (Diapharma, #K824094).

FVIII activity was inhibited by addition of 10 μg/ml of mouse monoclonal anti-human FVIII antibody clone RFF-VIIIC/8 (AbD Serotec, cat. # MCA4677). This antibody recognizes an epitope near the N-terminus of FVIII, does not react with VWF, and has been shown to inhibit FVIII activity. [27,28]

#### FVIII activity measurements in GMVEC and HUVEC lysates

Supernatants of GMVEC and HUVEC lysates and rFVIII standards were diluted in 1% BSA/PBS and prepared on ice. The FVIII activity standard curve was generated using rFVIII (Hexilate FS, 126 U/ml) diluted to produce activity values ranging from 0.6–40 mU/ml. The phospholipid/FIXa-FX mixture (1 part/5 parts) was also prepared on ice. All subsequent incubations occurred at 37°C with rotation. Sample volumes of 60μl, in duplicate, were added to clear 96-well plates, followed by 8 μl/well of phospholipid/FIXa-FX mixture. After mixing for 5 min, 100 mM CaCl_2_ was added (5μl/well, 5 mM final reaction concentration), and incubation was continued another 10 min. Following the addition of the chromogenic substrate, S-2765 + I-2581 (synthetic thrombin inhibitor) at 30μl/well, the incubation continued for 10 additional min. Absorbance was read at 405 nm and at 490 nm using a Tecan Infinite M200 plate reader. Subtraction of measurements at 490 nm corrected for absorbance differences between wells.

In FVIII activity inhibition assays, 10 μg/ml of mouse anti-human FVIII antibody (clone RFF-VIIIC/8) was added to samples of rFVIII with activity values ranging from 0.6–40 mU/ml, for 10 min on ice prior to assay commencement.

#### Statistical analysis

Probability (P) measurements were made using a 2-tailed Student’s t-test.

## Results

### The *F8* gene is expressed in GMVECs and HUVECs

Gene expression levels of *F8*, *VWF* and *AVPR2* (vasopressin V2 receptor gene) were measured in unstimulated GMVECs, HUVECs and fibroblasts by real-time PCR. The expression levels in each cell type were quantified relative to expression levels in GMVECs (Table 1 and S1 Dataset). The mRNA levels for *F8* and *VWF* are similar in GMVECs and HUVECs, whereas levels for *AVPR2* are 5-fold higher in GMVECs than in HUVECs. Fibroblast expression levels for *F8* are 6-fold higher and *AVPR2* levels are 3.5-fold higher than levels in GMVECs. Fibroblast mRNA levels for *VWF* are, however, ~20,000-fold lower than *VWF* message levels in GMVECs and HUVECs (Table 1). Threshold cycle numbers are shown in S3 Table and the transcript copy numbers after normalization to *GAPDH* are in S4 Table.

**Table 1:** Quantification of *F8*, *VWF* and *AVPR2* gene expression levels in GMVECs, HUVECs and fibroblasts (relative to GMVEC levels).

**GMVECs**	*F8*	*VWF*	*AVPR2*
Relative amounts (range)	1 (0.9–1.1)	1 (0.9–1.2)	1 (0.5–1.9)
**HUVECs**	*F8*	*VWF*	*AVPR2*
Relative amounts (range)	1.13 (1.0–1.2)	0.64^a^ (0.6–0.8)	0.20^a^ (0.1–0.3)
**Fibroblasts**	*F8*	*VWF*	*AVPR2*
Relative amounts (range)	6.20 (5.8–6.6)	5.1(10^−5^)^a^ (3(10^−5^)– 7(10^−5^))	3.51 (2.8–4.4)

RNA was extracted from untreated GMVECs, HUVECs and fibroblasts maintained in serum-free medium for 24 hours. The mRNA levels of *F8*, VWF, and *AVPR2* were quantified relative to the mRNA levels in GMVECs after normalization to *GAPDH* in each PCR analysis. Data were collected from 4–7 separate RNA extractions using each cell type.

^a^Indicates down-fold expression levels; 0.64 = 1.5 down-fold, 0.20 = 5 down-fold, and 5.1 (10^−5^) = 19,607 down-fold.

### Specificity of antibodies to human FVIII and VWF

**Fig 1:**
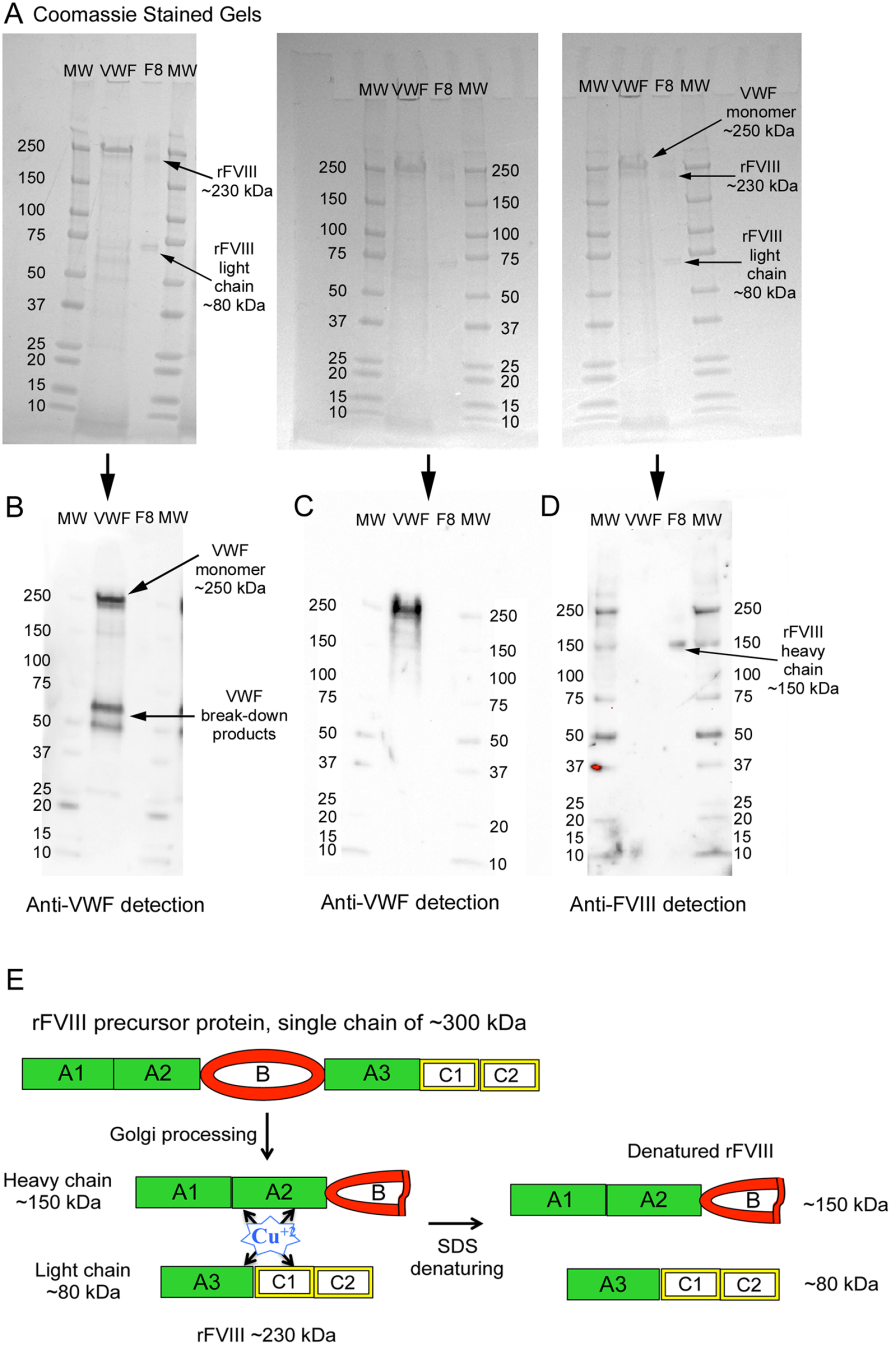
Specificity of antibodies to human FVIII and VWF. Denatured, non-reduced samples of recombinant (r) FVIII [Helixate FS, 130 ng per lane (1.30 IU)] and denatured, reduced samples of plasma purified VWF (90–130 ng per lane) were separated by 4–15% sodium dodecyl sulfate (SDS)-PAGE. Lanes containing rFVIII are marked as F8 and MW indicates molecular weight markers in kDa. (A) Arrows on the Coomassie stained gels show the ~230 and ~80 kDa bands for rFVIII and the monomer subunit of reduced VWF at ~250 kDa. (B, C and D) Western blots of gels shown in (A) were detected in (B) with goat anti-human VWF plus donkey anti-goat-HRP, in (C) with rabbit anti-human VWF plus donkey anti-rabbit-HRP, and in (D) with mouse monoclonal anti-human FVIII plus goat anti-mouse-HRP. Panel (E) is a schematic drawing illustrating the interpretation of the Coomassie stained bands and anti-FVIII detected bands generated from denatured rFVIII. The addition of SDS to rFVIII results in the dissociation of copper (Cu) ions [30,31] (or other ions that may be involved, such as the calcium and manganese ions required for FVIII activation) that bridge the heavy chain (~150 kDa) and light chain (~80 kDa) of the rFVIII protein (~230 kDa). [1] The rFVIII was produced using a ~90 kDa B domain. (The B domain was cleaved within the Golgi of the producing BHK cells prior to processing and metal coordination, resulting in a heavy chain of ~150 kDa.) [29].

Similar amounts of non-reduced recombinant (r) FVIII protein and reduced purified plasma VWF were analyzed by Western blotting using mouse monoclonal anti-human FVIII and polyclonal rabbit and goat anti-human VWF antibodies. Bands for both proteins were visible on Coomassie stained gels (Fig 1A). The mouse monoclonal antibody, produced using purified human FVIII as the immunogen, did not detect plasma-purified VWF (Fig 1D). The polyclonal anti-VWF antibodies did not detect the rFVIII protein (Fig 1B and 1C). The schematic drawing in panel (E) shows the generation of the rFVIII bands detected on the Coomassie stained gel and Western blot (Fig 1D). [29]

### Fluorescent microscopy emission “cross-talk” controls

**Fig 2:**
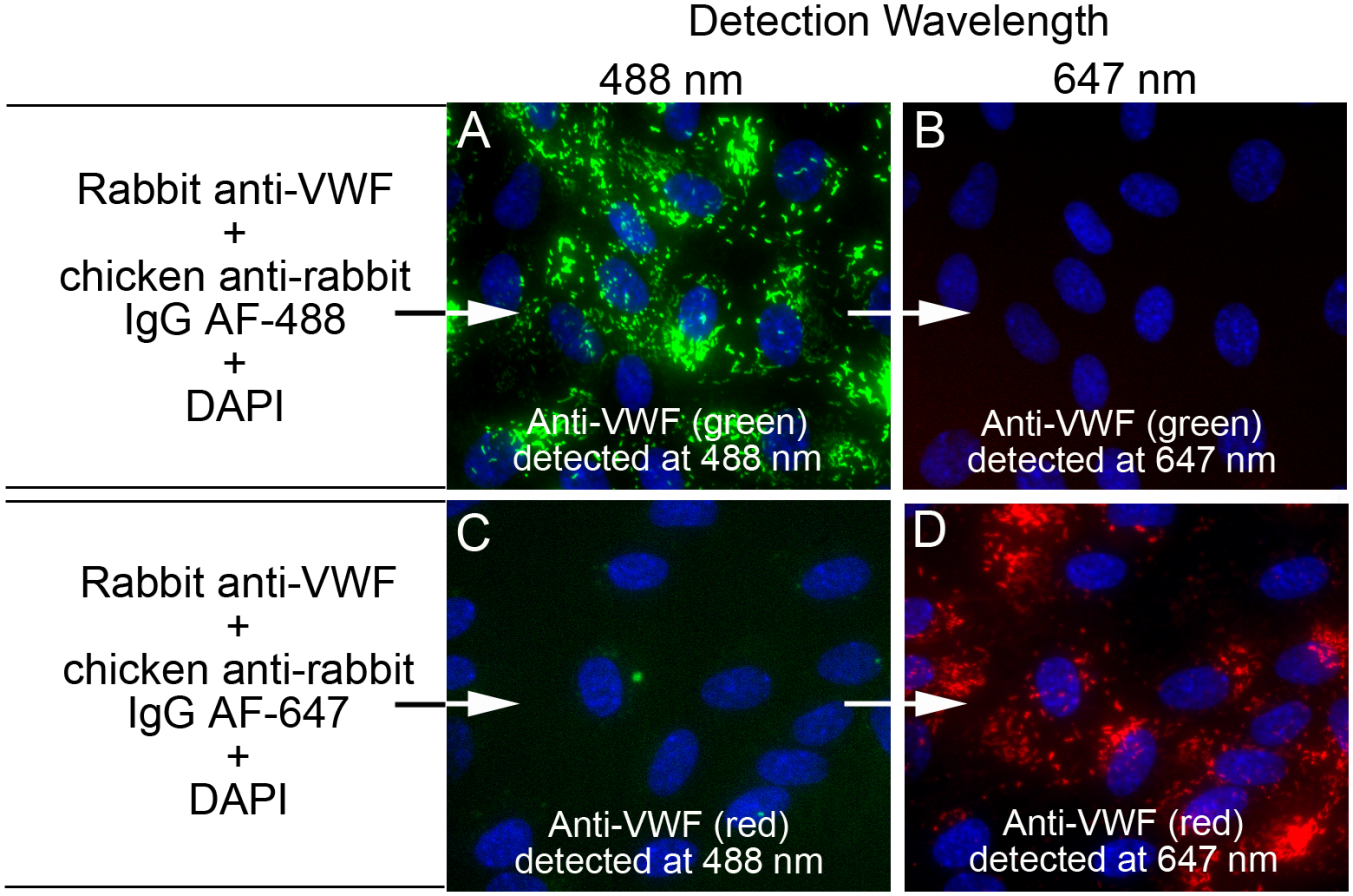
Fluorescent emission “cross-talk” controls. The high concentration of VWF present in Weibel-Palade bodies (WPBs) was used to demonstrate that images detected at the first wavelength channel (488 nm) were not cross contaminated by fluorescence generated from the second wavelength channel (647 nm), and reciprocally, the 647 nm channel was not affected by fluorescence from the 488 nm channel. Un-stimulated HUVECs were fixed with p-formaldehyde and treated with Triton-X to allow intracellular fluorescent staining. Cell nuclei were stained with DAPI (blue) and cells were imaged with a 60× objective. In (A and B) HUVECs on coverslip 1 were stained with rabbit anti-VWF plus chicken anti-rabbit IgG Alexa Fluor (AF)-488 (green) and in (C and D) HUVECs on coverslip 2 were stained with rabbit anti-VWF plus chicken anti-rabbit IgG AF-647 (red). Both coverslips were imaged at 488 nm and at 647 nm and merged with DAPI images. The images in (A) and (C) were detected at 488 nm and the images in (B) and (D) were detected at 647 nm.

The experiments shown in Fig 2 were conducted to verify the absence of fluorescent “cross-talk” or “bleed-through” between the channels used to detect and distinguish the proteins in this study by fluorescent microscopy: 488 nm (green) and 647 nm (red). Images of HUVECs internally stained only with rabbit anti-VWF + chicken anti-rabbit IgG AF-488 (green, Fig 2A) acquired using 647-channel filters did not show detectable 647 fluorescence (Fig 2B). HUVECs stained only with rabbit anti-VWF + chicken anti-rabbit IgG AF-647 (red, Fig 2D) acquired using 488-channel filters did not show any 488 fluorescence signal (Fig 2C).

### FVIII and VWF proteins are present in WPBs of GMVECs and HUVECs

**Fig 3:**
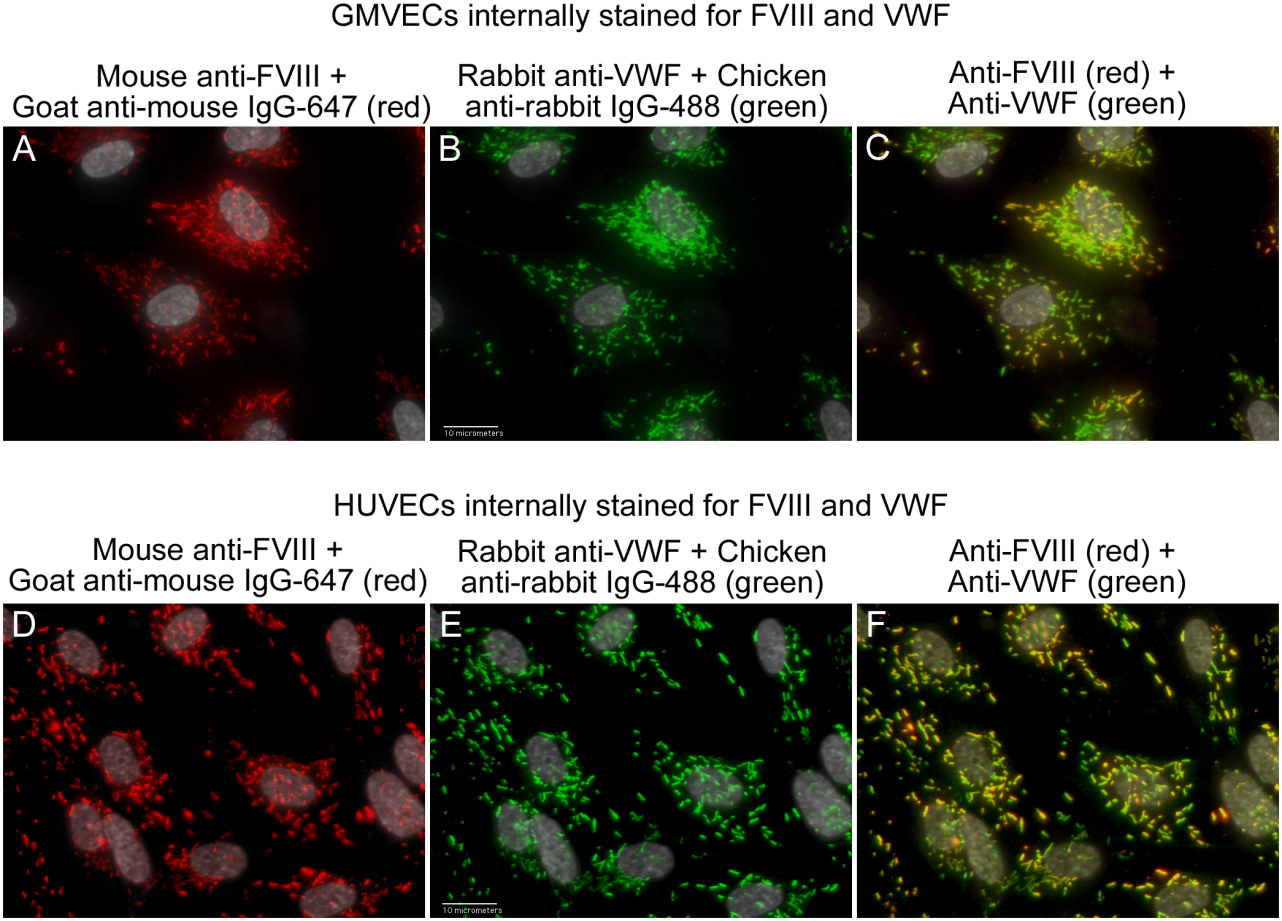
FVIII and VWF are present in the WPBs of GMVECs and HUVECs. Unstimulated GMVECs and HUVECs were fixed and treated with Triton-X to allow intracellular staining. Cells were then stained with mouse monoclonal anti-human FVIII plus goat anti-mouse AF IgG-647 (red), followed by staining with rabbit anti-human VWF plus chicken anti-rabbit AF IgG-488 (green). GMVEC images: (A) anti-FVIII detection (red); (B) anti-VWF detection (green); and (C) merged image of anti-FVIII plus anti-VWF. HUVEC images: (D) anti-FVIII detection (red); (E) anti-VWF detection (green); and (F) anti-FVIII plus anti-VWF. Images at 60× are shown merged with DAPI-detected nuclei (gray) and are representative of 5–7 experiments.

Internal staining of unstimulated GMVECs and HUVECs, using target-specific antibodies (Fig 1), demonstrates the presence of FVIII and VWF in WPBs (Fig 3 and S1 Fig). FVIII and VWF were distinctly identified by using mono-specific primary antibodies combined with secondary detection antibodies with widely separated wavelengths for excitation and emission, as shown in Fig 2. [24,26] GMVECs and HUVECs stained with secondary detection antibodies alone showed only background fluorescence (S2 Fig).

### FVIII and VWF proteins are absent in fibroblasts

**Fig 4:**
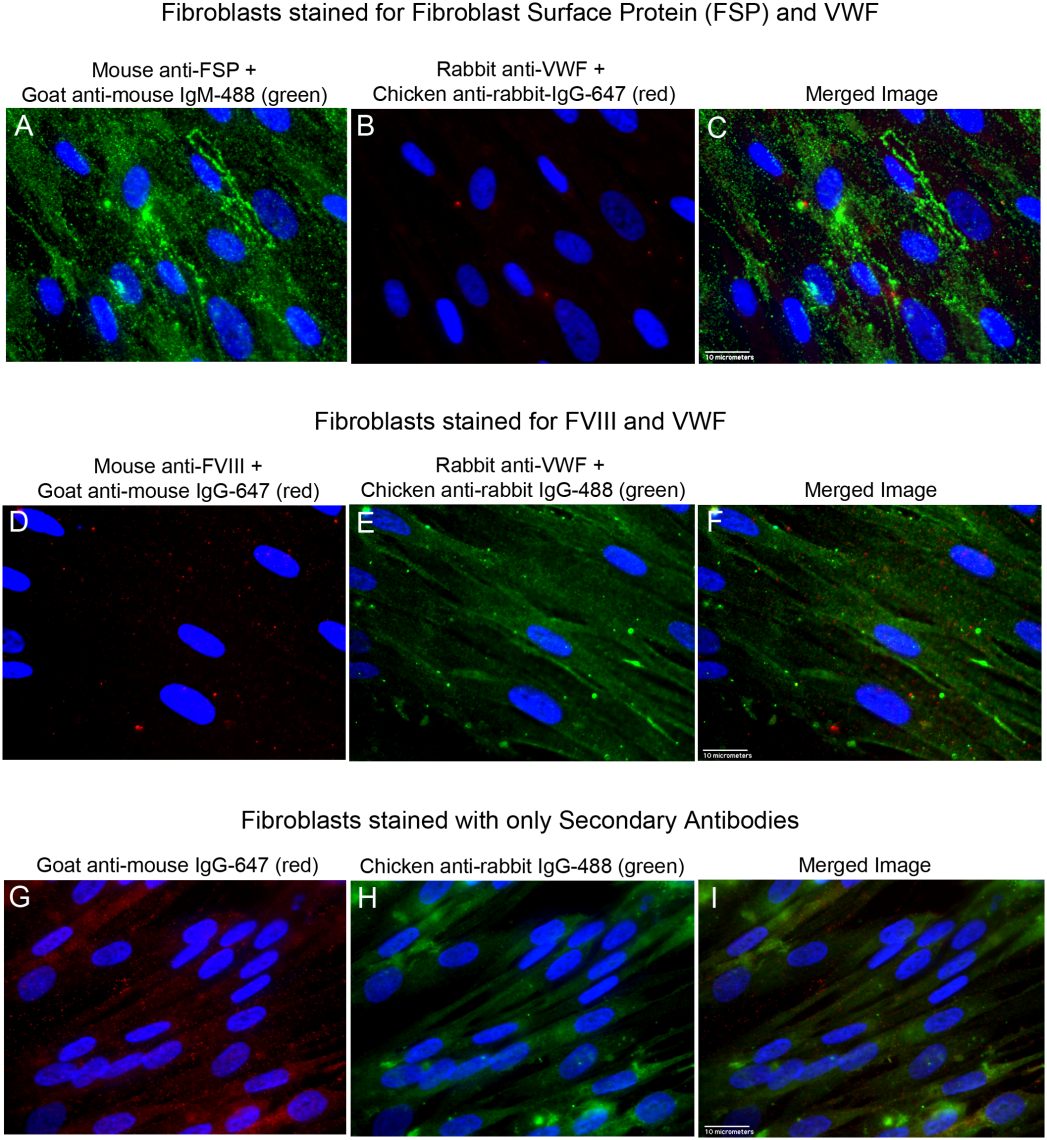
Fluorescent images of stained fibroblasts. Fibroblasts were stained with antibodies to Fibroblast Surface Protein (FSP), FVIII, and VWF plus relevant secondary antibodies, or with secondary detection antibodies alone. Images at 60× from each channel plus the merged image are shown. Cell nuclei were detected with DAPI (blue). (A-C) Fibroblasts were fixed and cell surfaces were stained with mouse anti-FSP plus goat anti-mouse IgM AF-488 (green). Cells were fixed again (to retain surface antibodies), and then treated with Triton-X for internal staining with rabbit anti-VWF plus chicken anti-rabbit IgG AF-647 (red). (D-F) Fibroblasts were fixed and treated with Triton-X for internal staining. Cells were stained with mouse anti-FVIII plus goat anti-mouse IgG AF-647 (red), followed by staining with rabbit anti-VWF plus chicken anti-rabbit IgG AF-488 (green). (G-I) Fixed and Triton-X-treated Fibroblasts were stained with only the secondary fluorescently labeled antibodies used in panels D-F for FVIII and VWF detection. Cells were stained with goat anti-mouse IgG AF-647 (red), and then with chicken anti-rabbit IgG AF-488 (green). Images are representative of 3 experiments.

In contrast to GMVECs and HUVECs, FVIII and VWF proteins were not detected by fluorescent microscopy in fibroblasts (Fig 4A–4F). The fibroblasts appeared to stain positively for VWF when the chicken anti-rabbit IgG-488 was used as the secondary detection antibody (Fig 4E and 4F), and negatively for VWF with chicken anti-rabbit IgG-647 as the secondary detection antibody (Fig 4B). Intensity measurements of full image areas of the fibroblasts were made after cell staining using each secondary detection antibody and in the presence or absence of primary antibodies to VWF. The intensity measurements showed that images of fibroblasts incubated with secondary IgG-488 antibodies alone had almost 2-fold higher fluorescent intensity than images of fibroblasts detected with the primary antibody to VWF plus secondary IgG-488 antibodies (S5 Table and S7 Dataset). Fibroblasts stained with secondary mouse antibodies alone also showed equal or higher amounts of fluorescent staining than fibroblasts stained with the primary mouse anti-FVIII plus these secondary detection antibodies (Fig 4D and 4G).

### Colocalization of FVIII and VWF in WPBs

**Fig 5:**
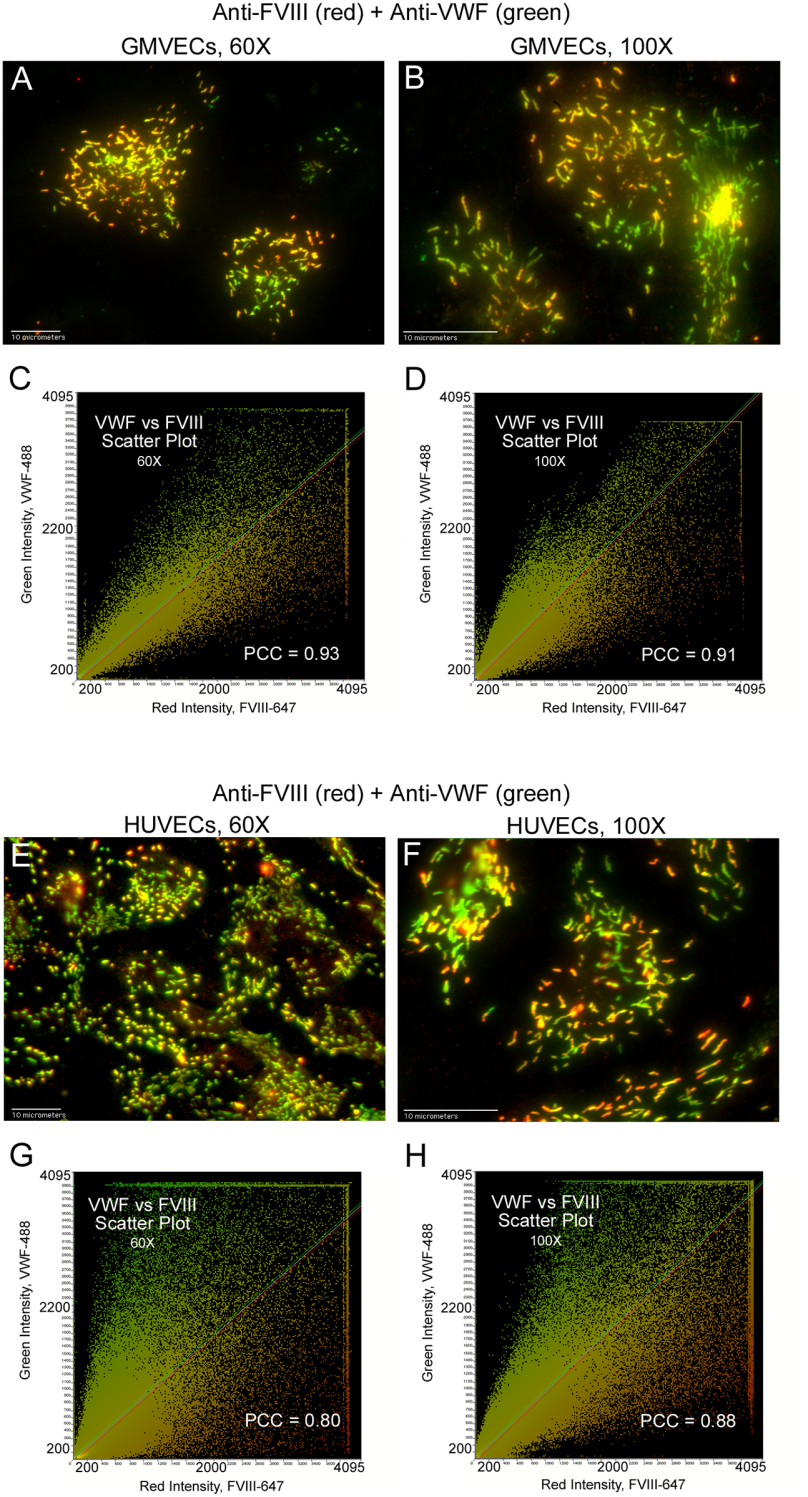
Statistical confirmation of the presence of FVIII and VWF in WPBs. GMVECs and HUVECs were treated with Triton-X to allow internal staining. Cells were then stained with mouse monoclonal anti-human FVIII plus goat anti-mouse IgG AF-647 (red) followed by staining with rabbit anti-human VWF plus chicken anti-rabbit IgG AF-488 (green). Merged images of FVIII and VWF detection, along with the corresponding intensity scatter plot, are shown in: (A and C) GMVECs at 60×, N = 4; (B and D) GMVECs at 100×, N = 5; (E and G) HUVECs at 60×, N = 7; and (F and H) HUVECs at 100×, N = 7. Values for Pearson’s correlation coefficient (PCC) are on each scatter plot.

Values for Pearson’s correlation coefficient (PCC) were calculated from merged fluorescent microscopy images of GMVECs and HUVECs with concurrent internal FVIII and internal VWF detection (Table 2, Fig 5 and S2 Dataset). The mean PCC values (0.77–0.79) resulting from FVIII and VWF detection in HUVEC and GMVEC images indicate: a correlation in location (WPBs); and a distribution of FVIII and VWF in proportional amounts within both types of ECs (Table 2). Intensity scatter plots, shown for representative images of FVIII and VWF in WPBs of GMVECs and HUVECs, show singular linear relationships (Fig 5C, 5D, 5G and 5H).

**Table 2:** Pearson’s correlation coefficients of internal FVIII in GMVECs and HUVECs concurrently stained with VWF.

	GMVECS	HUVECs
	FVIII and VWF, 60× Mean ± SD (range)	FVIII and VWF, 60× Mean ± SD (range)
**PCC**	0.77^a^ ± 0.09 (0.66–0.93)	0.79^a^ ± 0.05 (0.65–0.84)
	FVIII and VWF, 100**×** Mean ± SD (range)	FVIII and VWF, 100**×** Mean ± SD (range)
**PCC**	0.79^a^ ± 0.08 (0.65–0.92)	0.78^a^ ± 0.05 (0.65–0.87)

Pearson’s correlation coefficients (PCC) were measured in GMVEC and HUVEC images stained concurrently for FVIII and VWF. FVIII was detected using mouse anti- FVIII plus goat anti-mouse IgG AF-647 and VWF was detected with rabbit anti-VWF plus chicken anti-rabbit IgG AF-488. PCC values >0.5 signify detection correlation. [26] Data comparing FVIII and VWF were analyzed in 15–23 images from 4–5 experiments in GMVECs and in 27–29 images from 7 experiments in HUVECs.

^a^Indicates signal correlation in the two channels. [26]

### FVIII detection in WPBs does not overlap with detection of cytoplasmic proteins, β-actin or Factor H

**Fig 6:**
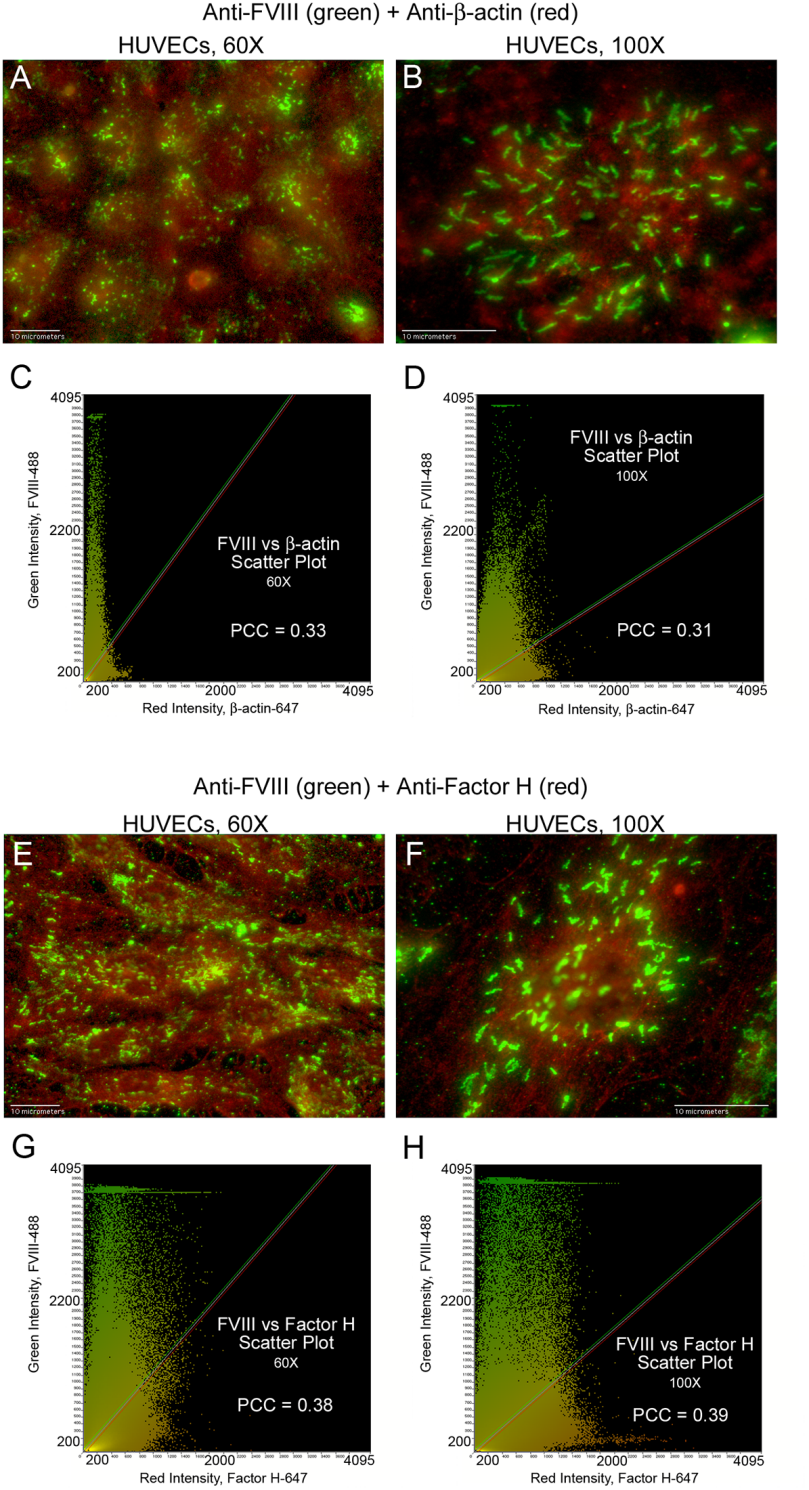
FVIII in HUVEC WPBs does not overlap with β-actin or Factor H. HUVECs were fixed and treated with Triton-X prior to staining with mouse monoclonal anti-FVIII plus donkey anti-mouse IgG AF-488 (green). This was followed by staining either with goat anti-β-actin plus chicken anti-goat IgG AF-647 (red) or with goat anti-Factor H plus chicken anti-goat IgG AF-647 (red). Merged images and the corresponding intensity scatter plots are: (A and C) FVIII and β-actin at 60×, N = 4; (B and D) FVIII and β-actin at 100×, N = 4; (E and G) FVIII and Factor H at 60×, N = 3; and (F and H) FVIII and Factor H at 100×, N = 3. Values for Pearson’s correlation coefficient (PCC) are on each scatter plot.

Fluorescent images of HUVECs with simultaneous detection of FVIII and either β-actin or Factor H, produced PCC values that were below the values for correlation (Table 3, Fig 6 and S2 Dataset). Mean PCC values for β-actin (0.35 and 0.38) and Factor H (0.42 and 0.44) indicate that detection signals for these two proteins are not spatially correlated with FVIII detection signals in HUVECs (Table 3). Intensity scatter plots display separate populations and the merged fluorescent images do not show FVIII in overlapping locations with β-actin or Factor H (Fig 6).

**Table 3:** Pearson’s correlation coefficients of internal FVIII in HUVECs concurrently stained with β-actin and Factor H.

	HUVECs	HUVECs
	FVIII and β-Actin, 60**×** Mean ± SD (range)	FVIII and Factor H, 60**×** Mean ± SD (range)
**PCC**	0.38^a^ ^,^ ^b^ ± 0.12 (0.02–0.5)	0.44^a^ ^,^ ^b^ ± 0.04 (0.38–0.5)
	FVIII and β-Actin, 100**×** Mean ± SD (range)	FVIII and Factor H, 100**×** Mean ± SD (range)
**PCC**	0.35^a^ ^,^ ^b^ ± 0.14 (0.0–0.5)	0.42^a^ ^,^ ^b^ ± 0.10 (0.31–0.62)

Pearson’s correlation coefficients (PCC) were measured in HUVEC images stained concurrently for FVIII and either with β-actin or with Factor H, using the antibody pairs described in the legend for Fig 6. Data comparing FVIII either with β-actin or with Factor H was analyzed from 12–14 images from 3–4 experiments of FVIII detection with each of these proteins.

^a^These values are below the PCC values >0.5 that signify detection correlation. [26]

^b^P<0.001 compared to PCC values obtained for FVIII detection correlated to VWF detection in HUVECs at both 60× and 100× (S2 Dataset).

### Intensity signals for FVIII and VWF in WPBs are synchronized

**Fig 7:**
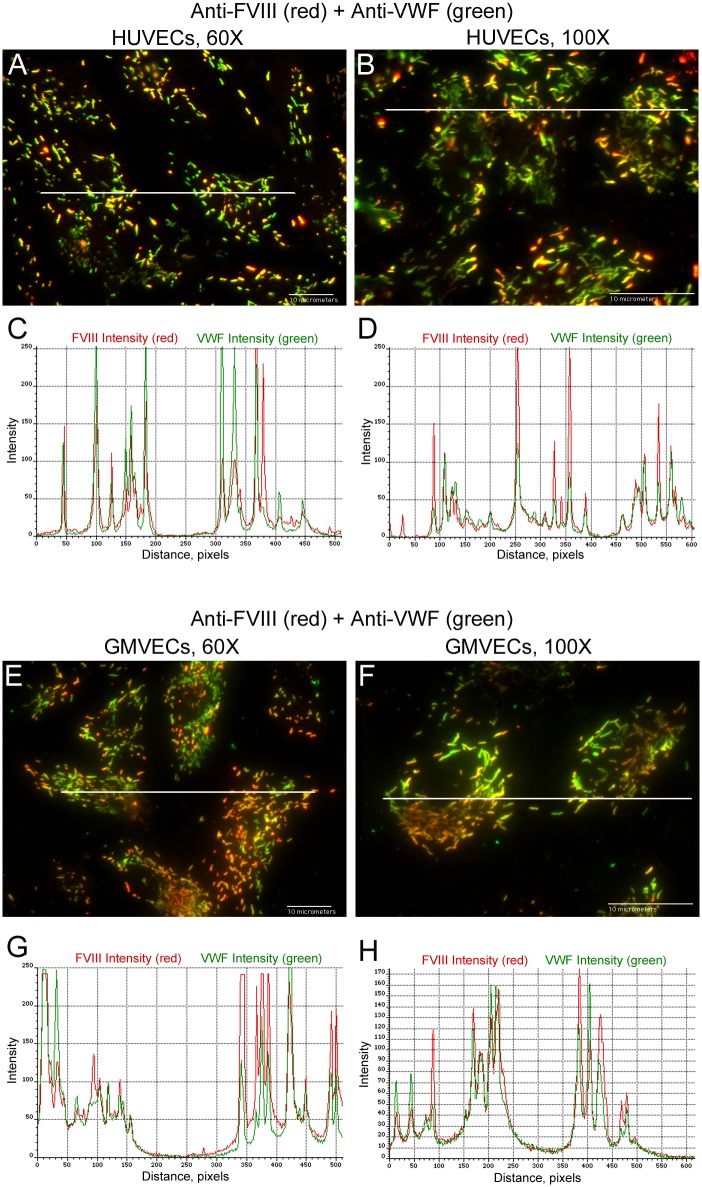
Intensity measurements of FVIII in WPBs synchronize with VWF intensities at identical locations. HUVECs (A and B) and GMVECs (E and F) were internally stained with mouse monoclonal anti-human FVIII plus goat anti-mouse IgG AF-647 (red) and with rabbit anti-human VWF plus chicken anti-rabbit IgG AF-488 (green). Graphs show the red (FVIII) and green (VWF) intensity values measured along a 68-μm line (from 60× images in graphs C and G) and a 35-μm line (from 100× images in graphs D and H) that traverses the WPBs. The data are representative of 5 experiments with GMVECs and 7 experiments with HUVECs.

Intensities were measured from merged fluorescent images of FVIII and VWF along lines traversing WPBs in internally stained HUVECs (Fig 7A–7D) and GMVECs (Fig 7E–7H). Intensity graphs of FVIII detection (red) and VWF detection (green) at the same locations demonstrate that the signals coincide (Fig 7 and S3 Dataset).

### Intensity signals of FVIII in WPBs are not synchronized with intensities of the cytoplasmic proteins β-actin or Factor H

**Fig 8:**
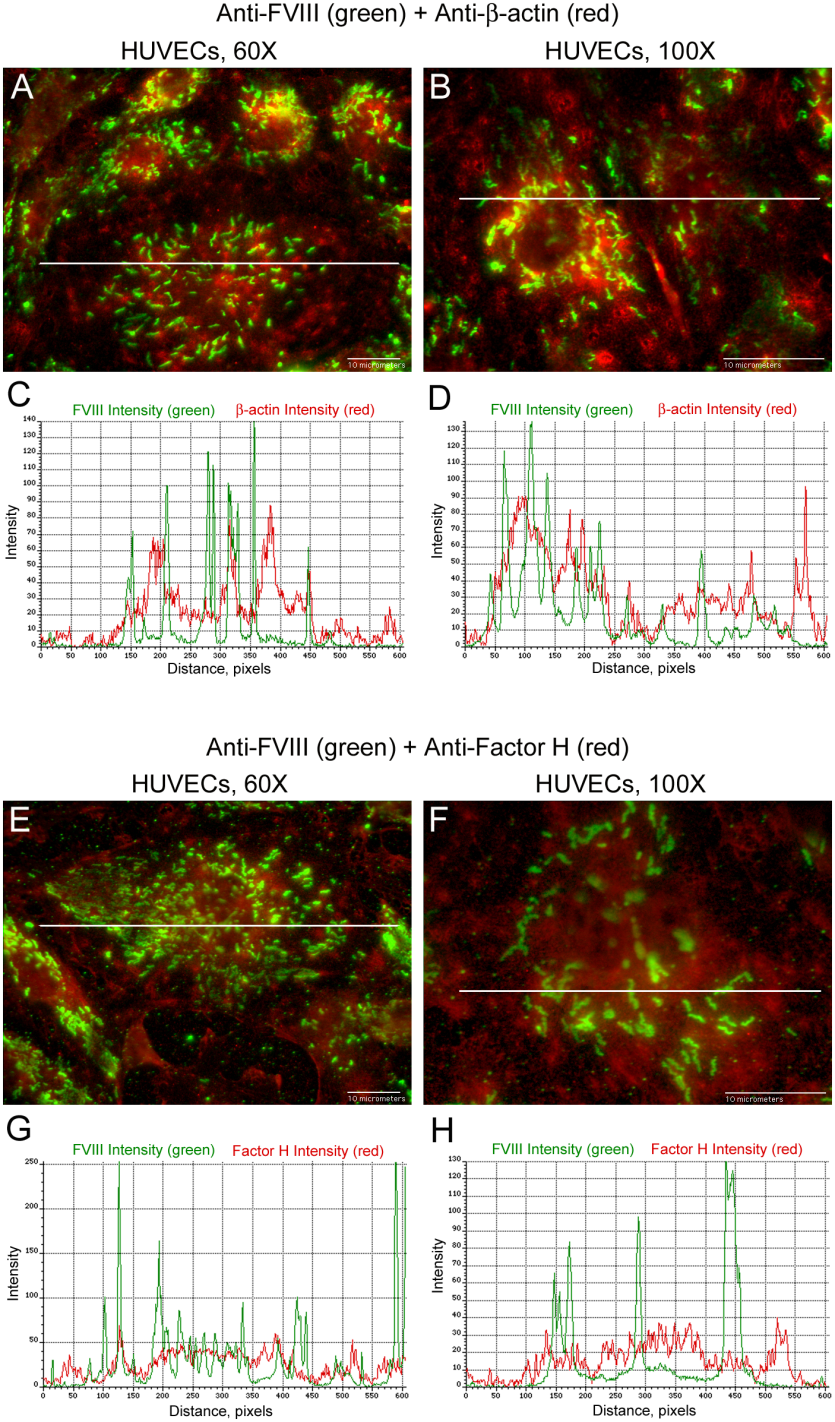
Intensity measurements of FVIII in HUVEC WPBs are not synchronized with intensities of β-actin or Factor H. HUVECs were internally stained with mouse monoclonal anti-human FVIII plus donkey anti-mouse IgG AF-488 (green), followed by staining either (A and B) with goat anti-β-actin plus chicken anti-goat IgG AF-647 (red) or (E and F) with goat anti-Factor H plus chicken anti-goat IgG AF-647 (red). Graphs below each merged image show the green intensity values of FVIII plus either: (C and D) the red intensity values of β-actin; or (G and H) the red intensity values of Factor H. The intensity values in the graphs were measured along lines that traverses FVIII detection in WPBs. Line lengths are 68 μm in 60× images and 35 μm in 100× images. N = 4 for FVIII measurement with β-actin and N = 3 for FVIII measurement with Factor H.

Contrary to FVIII and VWF detection, intensities measured along lines spanning WPBs in HUVECs internally stained with FVIII and concurrently with β-actin (Fig 8A–8D) or with Factor H (Fig 8E–8H) showed that the signals do not coincide (S4 Dataset).

### FVIII is secreted from stimulated GMVECs and HUVECs bound to ULVWF strings

**Fig 9:**
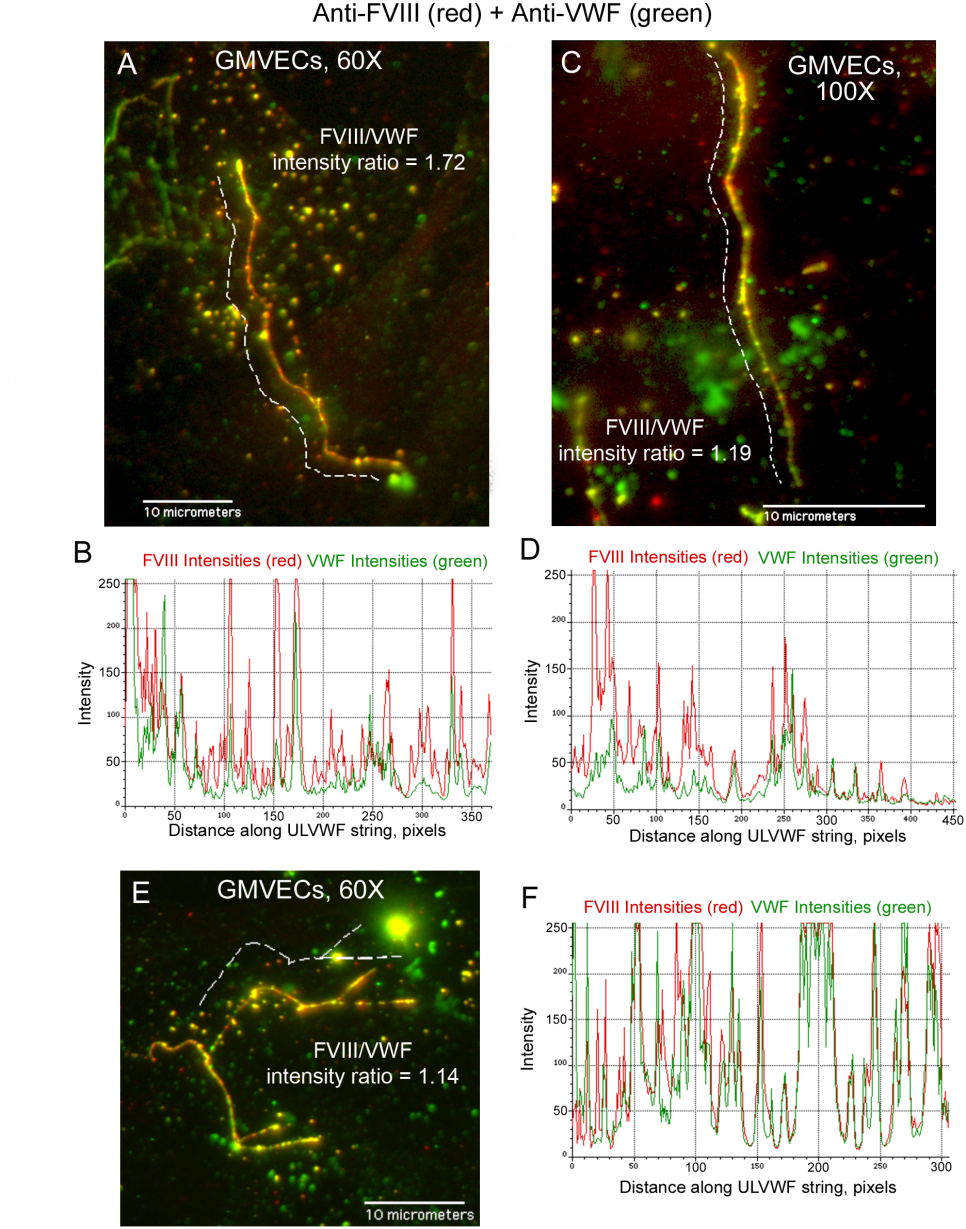
FVIII is secreted from stimulated GMVECs bound to ULVWF strings. GMVECs were stimulated with 100 μM histamine for 2 min. Cells were then stained with rabbit anti-VWF plus chicken anti-rabbit IgG AF-488 (green), washed and fixed. Following fixation, the cells were stained with mouse monoclonal anti-FVIII plus goat anti-mouse IgG AF-647 (red). Panels (A, C and E) show representative ULVWF strings with bound FVIII from merged images. Dashed lines (that were moved away to not obscure the string image) indicate the measured portions of the string. Corresponding graphs (B from image A, D from image C, and F from image E) show the intensities from the 488-nm (VWF, green) and 647-nm (FVIII, red) channels measured along the ULVWF string (in pixels) in the merged image. In the 60× images (A and E) 100 pixels = 11.4 μm and in the 100× image (C) 200 pixels = 11.8 μm. The ratio of FVIII intensity/VWF intensity is shown for each ULVWF string. Images are representative of 9–11 experiments.

**Fig 10:**
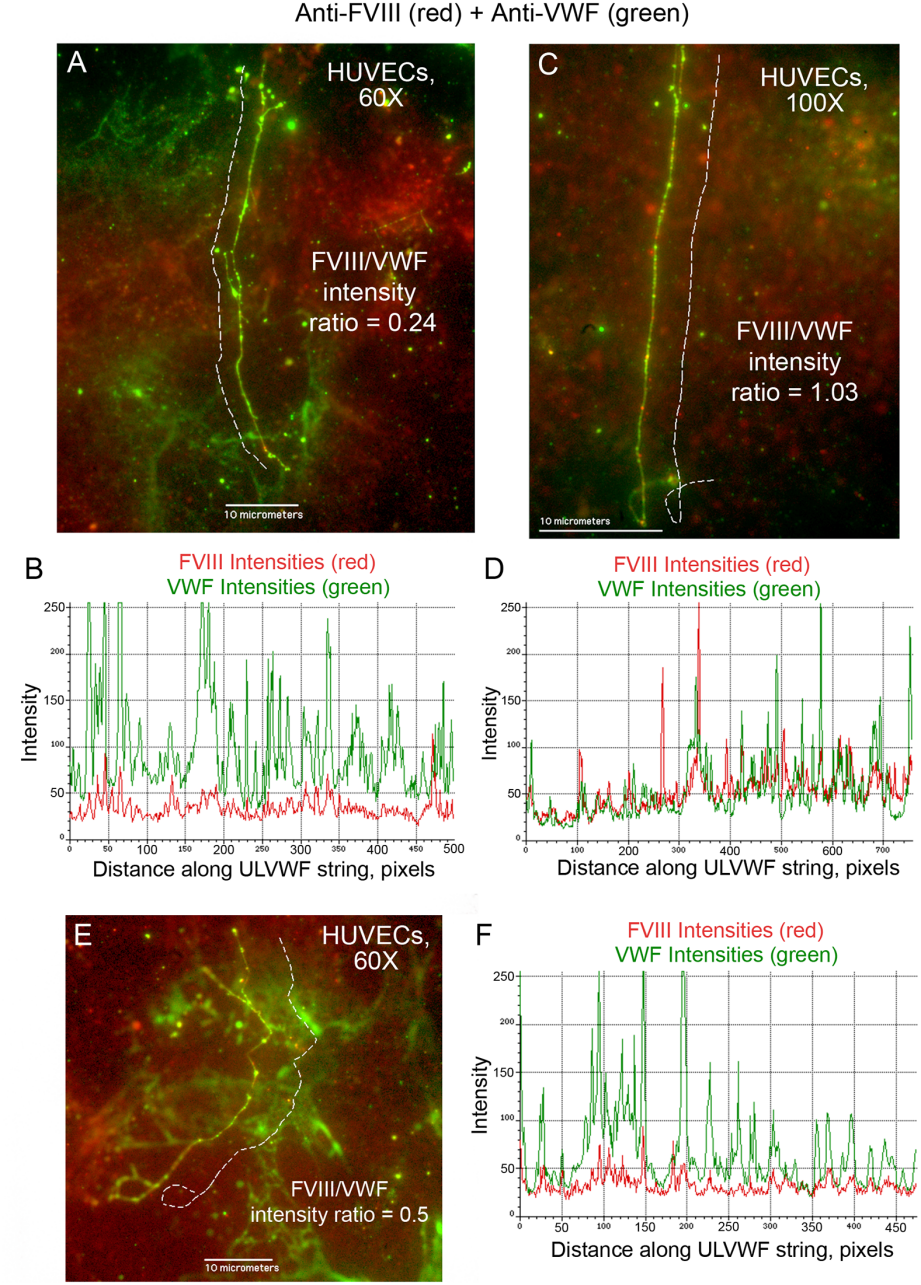
Less FVIII is bound to secreted/anchored ULVWF strings from stimulated HUVECs compared to GMVECs. HUVECs were stimulated with 100μM histamine for 2 min. Cells were then stained as described in the legend for Fig 9 with rabbit anti-VWF plus chicken anti-rabbit IgG AF-488 (green), and with mouse monoclonal anti-FVIII plus goat anti-mouse IgG AF-647 (red). Panels (A, C and E) show representative ULVWF strings with bound FVIII from merged images. The HUVEC-secreted/anchored ULVWF string in panel (A) has predominant VWF detection and further verifies the specificity of the VWF and FVIII antibodies and their distinct fluorescent signals. Dashed lines (that were moved away to not obscure the string image) indicate the measured portions of the string. Graphs (B from image A, D from image C, and F from image E) show the intensities from the 488-nm (VWF, green) and 647-nm (FVIII, red) channels measured along the ULVWF string (in pixels) from the corresponding merged image. In the 60× images (A and E) 100 pixels = 11.4 μm and in the 100× image (C) 200 pixels = 11.8 μm. The ratio of FVIII intensity/VWF intensity is shown for each ULVWF string. Images are representative of 4 experiments.

GMVECs and HUVECs stimulated with histamine resulted in WPB secretion from the two types of ECs. The EC-secreted and anchored ULVWF strings had bound FVIII (GMVECs Fig 9 and HUVECs Fig 10). The only possible source of this bound FVIII in these experiments was the EC WPB. Intensity measurements made along the secreted/anchored ULVWF strings from merged images demonstrate both FVIII and VWF. Intensity ratios of FVIII to VWF determined that ULVWF strings secreted by GMVECs have ~2-fold more FVIII attached than the strings secreted from HUVECs (Table 4, S5 and S6 Datasets).

**Table 4:** Intensity ratios of FVIII to VWF measured along EC-secreted VWF strings.

	String	FVIII	VWF	FVIII to VWF
	Lengths, μm (String #s)	Mean Intensity	Mean Intensity	Intensity Ratio (range)
**GMVECs 60×**	8–51 (45)	78.4 ± 35.2	83 ± 41.9	1.13 ± 0.6^a^ (0.23–2.7)
**GMVECs 100×**	4–27 (23)	79.6 ± 46.6	61.7 ± 33.3	1.4 ± 0.6^b^ (0.47–3.1)
**HUVECs 60×**	15–84 (15)	59.8 ± 60.2	91.4 ± 27.7	0.67 ± 0.6 (0.17–2.3)
**HUVECs 100×**	17–51 (14)	40.6 ± 16	75.5 ± 20.6	0.6 ± 0.3 (0.24–1.3)

Histamine-stimulated GMVECs and HUVECs were stained with rabbit anti-human VWF plus chicken anti-rabbit IgG AF-488, followed by staining with mouse FVIII plus goat anti-mouse IgG AF-647. Intensity values from the 488-nm and the 647-nm channels were measured along merged images of the EC-secreted and anchored ULVWF strings. Data (means ± SD) were collected from 4 HUVEC and 11 GMVEC experiments.

^a^Indicates P = 0.01 compared to HUVEC values at 60×

^b^Indicates P = <0.001 compared to HUVEC values at 100×

In contrast to stimulated ECs, there was only light detection of FVIII and VWF on surfaces of unstimulated HUVECs. (The HUVECs were perturbed only mechanically in washing steps.) Antibodies against FVIII and VWF detected only small amounts of surface FVIII and VWF in non-overlapping locations (S3 Fig).

### Internal FVIII activity in GMVECs and HUVECs

Internal FVIII activity was measured in the supernatants of GMVEC lysates and HUVEC lysates using a modified chromogenic FVIII activity assay (Table 5 and S8 Dataset). The specificity of the FVIII assay was verified by the reduced activity in rFVIII dilutions with addition of inhibitory FVIII antibodies (S4 Fig). Additional confirmation was shown by parallel reductions in FVIII activity values resulting from dilutions of EC lysates (S4 Fig and S8 Dataset).

**Table 5:** FVIII activities in GMVEC and HUVEC cell lysates.

	GMVECs	HUVECs
**FVIII activity mU/ml**	1.52 ± 0.74	2.57 ± 0.63
**Activity Ranges**	0.75–2.68	1.64–3.59
**Number of Flasks**	8	12

The FVIII activity was measured in the supernatants of GMVEC and HUVEC lysates using a modified chromogenic activity assay. The lysates were collected from ECs in T-75 flasks solubilized with 0.5 ml of mammalian cell lysis reagent. Values shown for FVIII activity are means ± standard deviations.

## Discussion

In this study, we measured the relative gene expression of *F8* and *VWF*, using real-time PCR, in two types of primary human ECs, GMVECs and HUVECs. This is the first report of *F8* gene analysis in GMVECs. Furthermore, our results contradict several previous studies that specifically reported the lack of *F8* gene expression in HUVECs.

Using a different experimental system, a microarray expression BeadChip, Dashty, et al., studied the gene expression of multiple coagulation factors in eight human primary cell types. [32] These included mesenchymal stem cells, hepatocytes, adipocytes, preadipocytes, monocytes, macrophages, HUVECs and dermal fibroblasts. These authors reported that HUVECs did not express mRNA for *F8*. All of the other cell types (including dermal fibroblasts) were reported to express similar levels of the *F8* gene (~30-fold higher than HUVEC levels). The HUVEC *F8* gene levels may have been below the threshold for positive detection in their system. We consider this likely because the findings by Dashty, et al., are not consistent with our findings (using real-time PCR) that the *F8* mRNA levels were expressed in the same order of magnitude in HUVECs, GMVECs and fibroblasts (S4 Table). We question the interpretation of the BeadChip analysis used by Dashy, et al. because they reported *VWF* gene expression, known to be present only in megakaryocytes, platelets and ECs, to be positive in every cell type used in their study (with the exception of adipocytes). In contrast, in our experiments, *VWF* expression levels in fibroblasts were 10,000-fold lower than *VWF* gene expression in HUVECs or GMVECs (S4 Table).

In an article, by Shahani, et al., *F8* gene expression was measured in HCMECs and HUVECs and, using real time PCR, quantified relative to the *F8* gene levels in their *F8*-transfected CHO cells. [13] The *F8* levels in their HCMECs were 93-fold higher that the *F8*-transfected CHO cells. The HUVEC *F8* gene levels were “0-fold” higher, apparently indicating that HUVECs expressed the same amount of *F8* mRNA as their *F8*-transfected CHO cells. The gene levels were evaluated using the 2^–ΔΔCT^ method (and 2^0^ = 1); therefore, the HUVECs and the *F8*-transfected CHO cells produced equal amounts of *F8* mRNA. Shahani, et al. concluded, however, that *F8* gene expression was not detected in HUVECs.

In a separate study by Shovlin, et al., their presentation of *F8* gene expression in cultured HUVECs is difficult to interpret. [14] In addition to the known *F8* transcripts variants 1 and 2, these authors detected two previously unknown *F8* mRNA transcripts (designated as variants 3 and 4). Transcripts for *F8* variants 1 or 2 were detected, by PCR cDNA gel analysis and sequencing, in each cultured EC type tested: human pulmonary (HP) arterial ECs, HP microvascular ECs, HDMECs and HUVECs. Their HUVECs were also positive for *F8* mRNA variant 4. Although the authors showed a gel of HUVEC cDNA clearly positive for 2 out of 4 of the *F8* mRNA transcripts studied, they used HUVECs as “negative controls” for FVIII antigen measurements and surface detection in flow cytometer experiments, and concluded that “in their hands, HUVECs were not a good source of FVIII.”

In two separate studies, the shapes of WPBs were examined in ECs transfected with the human *F8* gene.[33,34] In the 2009 study, by van den Biggelaar, et al., HUVECs transfected either with untagged or with *F8*-yellow-fluorescent-protein (YFP) were examined by fluorescent microscopy. [34] The untagged FVIII protein was detected using a monoclonal antibody to FVIII plus fluorescently tagged secondary antibody. These authors found that HUVEC WPBs were in the usually observed elongated shape in the absence of *F8* gene transfection, and spherical in shape with either untagged *F8* or *F8*-YFP transfection. van den Biggelaar, et al. concluded that endogenous FVIII production could interfere with normal folding of VWF into tubular forms; however, it was not determined what levels of FVIII production would have this effect on the WPBs. In the 2011 study, Bouwens, et al., transfected blood outgrowth endothelial cells (BOECs) with *F8*-green-FP (GFP), and analyzed WPB shape and structure using correlative light and electron microscopy (CLEM). [33] The authors made detailed length and width measurements of FVIII positive and FVIII negative WPBs, and concluded that WPBs containing FVIII had diameters 1.5-fold larger than WPBs without FVIII. They also concluded that the observed structural changes in the WPBs of the *F8*-transfected BOECs might be due to over expression of FVIII protein in these cells.

Using fluorescent microscopy, in the current study, we showed that FVIII was stored together with VWF in GMVEC and HUVEC WPBs. Their concomitant location in WPBs was confirmed by three means: direct visual evidence in merged fluorescent images; synchronized signal detection for both proteins across merged WPB images; and calculated correlation coefficients. In a recently published article, we also used fluorescent microscopy and correlation coefficient measurements to establish that Factor H and Factor I are not located in WPBs of GMVECs and HUVECs. [35]

Our current methods for detection of FVIII in HUVEC (and GMVEC) WPBs, is possible, in comparison to studies of cellular FVIII in in the 80’s and 90’s, because of advances in microscopy, detection sensitivity, and availability of monoclonal antibodies against FVIII. Our current detection methods also differ from other contemporary studies of FVIII synthesis in cultured ECs. [11–14] For example, the study conducted by Shahani, et al. in 2010, employed laboratory methods that were similar to the methods we used in this report, except with crucial differences, to detect internal FVIII protein, measure cell-released and cell lysate FVIII activity, and *F8* gene expression levels in primary cultured human ECs. [13] As mentioned above in the third paragraph of the Discussion, Shahani, et al. found that HUVEC *F8* gene levels were about 100-fold lower than the *F8* gene levels in HCMECs, and were equal to levels in their *F8*-transfected CHO cells. Although HCMEC gene levels of *F8* could possibly be 100-fold higher than those in HUVECs, there are several potential technical reasons that may account for their results: RNA was extracted from a relatively small number of ECs grown in 6-well plates (with growth areas of 9.6 cm^2^); RNA quality and quantity were not verified by gel analysis; results were based on a single RNA extraction per cell type; and PCR primers were designed in their laboratory (in contrast to our commercially tested and guaranteed TaqMan probes). In our studies, we used TaqMan probes and RNA was extracted 4–7 times from each EC type (or fibroblasts) grown in T-75 flasks (with growth areas of 75 cm^2^ and ~7-fold more cells than in the experiments of Shahani, et al.). In addition, we extracted RNA from ECs when the majority of cells were fully mature, but not overgrown (HUVECs: 7–14 days post-seeding; and GMVECs: 11–23 days post-seeding). We quantified RNA by optical density, RNA integrity was verified by gel analysis, and gene expression levels were measured in triplicate from 3–7 PCR runs. Gene expression levels for *F8* were calculated relative to expression levels in GMVECs, a natural cell type, instead of the non-standardized laboratory transfected cell line used by Shahani, et al. [13]

As in our study, Shahani, et al. used monoclonal antibodies directed against FVIII and polyclonal antibodies against VWF, combined with fluorescent secondary antibodies, to detect internal FVIII in cultured ECs. [13] Their images of HUVECs co-stained for FVIII and VWF showed no detectable FVIII; however the fluorescence detection of VWF in WPBs was also low. Several differences between their staining method and ours are likely to contribute to the low fluorescent signals in their images: their ECs were grown, stained and imaged on chamber slides, and the thicker glass compromises fluorescent signals; and, although their ECs were stained after reaching confluence, the number of days post-confluence was not stated. There is, therefore, the possibility that their ECs were immature and not capable of packaging WPBs with FVIII and VWF. Another problem with the studies of Shahani, et al., may be the length of time between staining and imaging of the ECs. Their cells were stained and then stored for an unstated time at negative 20°C until imaging. Fluorescent signals decrease over time, even if slides are stored in the dark at low temperatures. [13] In contrast, in our study cells were grown, stained and imaged on 1-mm thick coverslips; stained no earlier than 6 days after seeding; and imaged on the same day as staining. Furthermore, we include experiments specifically designed to demonstrate that fluorescent detection antibodies did not cause any “bleed-through” or “cross-talk” in our microscopic system, and provide control images of each cell type stained with secondary antibodies alone.

Stimulation of GMVECs and HUVECs causes WPB secretion, and we found that the secreted/anchored ULVWF strings were bound with FVIII. Measured intensities of these proteins revealed that the GMVEC-secreted ULVWF strings carried more FVIII than the HUVEC-secreted ULVWF strings. This observation of FVIII bound to EC-secreted ULVWF strings has been previously reported by Bouwens, et al., using blood outgrowth ECs transfected with *F8*. The *F8*-transfected ECs produced *F8*/ULVWF strings with an unexpected characteristic; a decrease in the ability of the *F8*/ULVWF strings to bind platelets compared to the un-transfected outgrowth ECs. The loss of the platelet adhesive capacity of these *F8*/ULVWF strings was attributed to a steric hindrance affect from FVIII binding nearby to platelet binding sites within A1 domains of VWF. [33] This explanation for their observation is unlikely because ULVWF strings secreted from normal GMVECs and HUVECs [36] actively bind platelets and are naturally bound with FVIII, as we show in the current study.

VWF multimers are produced, stored and secreted in long, hyper-adhesive strings bound with FVIII from endothelial cell WPBs. These FVIII bound VWF multimeric strings are transiently anchored to EC surfaces. ADAMTS-13, the VWF cleaving protease, cleaves the anchored VWF strings into smaller, soluble forms that bind FVIII and prolong FVIII survival in the circulation. [22]

V2 receptors must be present on EC surfaces in order for cyclic AMP-mediated signaling to induce FVIII and VWF secretion in response to DDAVP administration. [37,38] Transcripts for the vasopressin V2 receptor gene, *AVPR2*, have been found in human lung MVECs and in other EC-containing tissues, although not in HUVECs. [38,39] In one of these previous reports, HUVECs transfected with *AVPR2* were shown to secrete measurable amounts of VWF after combined stimulation of DDAVP plus the phosphodiesterase inhibitor, 3-isobutyl-1-methyl-xanthine (IBMX). [39] We also measured relative levels of *AVPR2* in addition to the gene expression of *F8* and *VWF* in GMVECs and HUVECs. Our ECs expressed low levels of *AVPR2* (S1 and S2 Tables), probably leading to the presence of some V2 receptors on their (and fibroblast) surfaces.

In this study, we found that fibroblasts have higher *F8* mRNA transcript levels than GMVECs and HUVECs, (and very low levels of *VWF*), although neither FVIII protein nor VWF protein was detected by fluorescent microscopy. Cells unable to synthesize sufficient amounts of VWF do not form WPBs, and only a few cell lines transfected with *VWF* have been shown to store the induced VWF into organelles resembling WPBs. [40,41] Furthermore, non-VWF producing cells transfected with *F8* do not store FVIII protein nor form WPBs, whereas HUVECs transfected with the *F8* gene have been shown to produce and store FVIII in WPBs with native VWF. [42,43] Rosenberg et al. has also demonstrated that only cells doubly transfected with genes for *F8* and *VWF* results in the storage of both proteins in WPBs. [43] This finding is analogous to the FVIII/VWF production in Type 3 VWD, when the near absence of VWF protein also results in a near absence of FVIII protein. [16]

A sensitive and widely used method of measuring the low levels of FVIII activity in cultured cells uses a chromogenic substrate. [10–13,44] The chromogenic substrate is cleaved into a detectable colored product by activated FXa, which is generated from FX by FIXa in complex with FVIII (the limiting factor that is measured). The FXa generated and the intensity of the cleaved substrate is, therefore, proportional to the FVIII available.[32] Shahani, et al. did not detect FVIII activity in cell lysates of HUVECs grown in 96-well plates. In contrast, in our study we increased the ratio of cells to the volume of lysis buffer, and modified the chromogenic assay to accommodate a larger sample. With these alterations, we were able to measure FVIII activity in HUVEC lysates, as well as in GMVEC lysates.

Our results demonstrate that stimulated GMVECs and HUVECs secrete cell-anchored ULVWF multimeric strings covered with bound FVIII. There is no established method to quantify EC-secreted FVIII activity that is attached to ULVWF strings. Furthermore, under our experimental conditions, ADAMTS-13 (also released from ECs) cleaves EC-anchored ULVWF strings; but it is not known if string-bound FVIII remains attached to smaller VWF cleavage products, dissociates from cleaved VWF forms, becomes free in solution, or binds to EC surfaces. In order to avoid these complicating and enigmatic issues in the current study, we measured FVIII activity in the cell lysates of PBS-washed GMVECs and HUVECs. The use of EC lysates also avoided any inclusion of exogenous bovine FVIII activity from culture medium supplements in our measurements.

In conclusion, our data provides new information about human FVIII and VWF obtained by using two types of human, unmodified ECs. These genetically unaltered GMVECs and HUVECs have transcripts for both *F8* and *VWF*, produce both FVIII and VWF proteins, store both proteins together in WPB organelles, and secrete them both in response to EC stimulation. This new information, which supports previous reports in human cultured ECs [11–13] and more recent findings in mice, [19,20] may lead to improved therapy for hemophilia A. Recognition that FVIII is normally synthesized in human ECs and stored in their WPBs for regulated secretion (along with VWF) indicates that these organelles may ultimately become the precise, most effective physiological targets for human *F8* gene delivery.

## Supporting Information

S1 Dataset Gene expression data and calculations. Gene expression levels of *F8*, VWF and *AVPR2* were measured in unstimulated GMVECs, HUVECs and fibroblasts by real time PCR. The expression levels in each cell type were quantified relative to expression levels in GMVECs.
(XLSX)

S2 Dataset Correlation coefficients data. Values for Pearson’s correlation coefficient (PCC) were calculated from merged fluorescent microscopy images of GMVECs and HUVECs with concurrent internal FVIII and internal VWF detection and in HUVECs with simultaneous detection of FVIII and either β-actin or Factor H.
(XLSX)

S3 Dataset Graphs of FVIII and VWF signal intensities measured across GMVEC and HUVEC WPBs. Graphs produced from intensities measured from merged fluorescent images of FVIII and VWF along lines traversing WPBs in internally stained HUVECs and GMVECs.
(XLSX)

S4 Dataset Graphs of FVIII and β-actin or FVIII and Factor H signal intensities measured across HUVEC WPBs. Graphs produced from intensities measured along lines spanning WPBs in HUVECs internally stained with antibodies to FVIII and concurrently with anti-β-actin or with anti-Factor H.
(XLSX)

S5 Dataset Intensity data for FVIII and VWF measured along ULVWF strings. Intensity measurements made along the GMVEC and HUVEC secreted/anchored ULVWF strings from merged images stained with antibodies to FVIII and VWF.
(XLSX)

S6 Dataset Graphs of FVIII and VWF signal intensities measured along ULVWF strings. Graphs produced from intensities measured along GMVEC and HUVEC secreted/anchored ULVWF strings from merged images stained with antibodies to FVIII and VWF.
(XLSX)

S7 Dataset Intensity data of fibroblasts after fluorescent immunostaining. Intensity measurements were made on full image areas of fibroblasts after these cells were stained using each secondary detection antibody alone and in the presence of primary antibodies to VWF.
(XLSX)

S8 Dataset FVIII activity data measured in GMVECs and HUVECs. FVIII activity was measured in GMVEC and HUVEC cell lysates and in dilutions of rFVIII with and without inhibitory antibodies using a chromogenic Coatest FVIII activity assay.
(XLSX)

S1 Fig FVIII and VWF are shown to be present in WPBs of HUVECs using a second set of detection antibodies. Unstimulated HUVECs were fixed with 1% p-formaldehyde and treated with Triton-X to allow intracellular staining. Cells were stained with mouse monoclonal anti-human FVIII plus chicken anti-mouse IgG AF-647 (red), followed by staining with goat anti-human VWF plus donkey anti-rabbit IgG AF-488 (green). The mouse monoclonal antibody to FVIII is the same one that was used throughout the study. This primary polyclonal goat VWF antibody and both secondary detection antibodies were used only in this set of fluorescent detection experiments. The goat anti-VWF was also used for Western blot detection in Fig 1. The HUVEC images are at 60X: (A) anti-FVIII detection (red); (B) anti-VWF detection (green); and (C) merged image of anti-FVIII plus anti-VWF. Panel D is the intensity scatter plot of the merged image in (C) with the colocalization coefficient (PCC) value that is described later in this article.
(TIF)

S2 Fig GMVECs and HUVECs stained with only Alexa Fluor (AF)-labeled secondary antibodies. GMVECs (A-C) and HUVECs (D-F) were treated with Triton-X to allow internal staining. Cells were then stained with goat anti-mouse IgG AF-647 (A and D, red) and chicken anti-rabbit IgG AF-488 (B and E, green) secondary detection antibodies at final concentrations of 20 μg/ml before mounting and image acquisition at 60×. Cell nuclei were detected with DAPI (blue).
(TIF)

S3 Fig Surfaces of unstimulated HUVECs stained with antibodies to FVIII and VWF. HUVECs were washed and fixed before surfaces were stained with mouse anti-FVIII + goat anti-mouse IgG AF-647 and rabbit anti-VWF + chicken anti-rabbit IgG AF-488. Single channel detection images are shown in (A) mouse anti-FVIII (647, red), and in (B) rabbit anti-VWF (488, green), with the merged image in (C).
(TIF)

S4 Fig Specificity of FVIII activity assay is demonstrated by antibody inhibition and sample dilutions. (A) FVIII activities, ranging from 0.6–10 mU/ml, in dilutions of rFVIII, were inhibited by addition of 10 μg/ml mouse anti-human FVIII antibody (clone RFF-VIIIC/8) for 10 min (on ice) prior to the start of the chromogenic Coatest assay. The green triangles represent rFVIII without antibody addition and the red squares and blue diamonds are 2 separate rFVIII dilutions with final concentrations of 10 μg/ml anti-FVIII. (B) FVIII activity was measured in HUVEC cell lysates diluted 1.3-, 2-, 3- and 4-fold in 1% BSA/PBS.
(TIF)

S1 Table TaqMan Gene Expression Assay Probes. PCR amplified products were detected using TaqMan Gene Expression Assays with 6-carboxyfluorescein-labeled probes that span target exon junctions (Life Technologies).
(PDF)

S2 Table Alexa Fluor (AF)-labeled secondary antibodies. The AF-labeled fluorescent secondary antibodies were supplied at 2 mg/ml and were used at a final concentration of 20 μg/ml in 1% BSA/PBS (Life Technologies).
(PDF)

S3 Table Threshold cycle numbers for each cell type. Average number of PCR cycles ± standard deviation to reach the threshold fluorescent signal detection in real-time PCR using TaqMan probes. Data are from 4–7 separate RNA isolations from each cell type. Threshold cycle numbers are means from 3–7 PCR analyses with triplicate measurements within each PCR experiment.
(PDF)

S4 Table Comparison of mRNA copy numbers. The average mRNA copy numbers for each cell type at the threshold cycle (C_T_ shown in S3 Table) was normalized to the mean copy number of *GAPDH* for each cell type at the threshold cycle. The copy number = 2^-CT^.
(PDF)

S5 Table Fluorescent intensities of fibroblasts stained with secondary detection antibodies with and without primary antibodies to VWF. Fluorescent intensities were measured in fibroblasts internally stained separately with rabbit anti-VWF plus two different secondary detection antibodies or with these secondary detection antibodies alone. Staining with secondary chicken anti-rabbit IgG-488 resulted in higher fluorescent intensities than rabbit anti-VWF + chicken anti-rabbit IgG-488.
(PDF)
