# Supplementary figures and images for: Factor VIII Is Synthesized in Human Endothelial Cells, Packaged in Weibel-Palade Bodies and Secreted Bound to ULVWF Strings
Source: PLoS One. 2015 Oct 16;10(10):e0140740. doi: 10.1371/journal.pone.0140740 (PMC4608722; doi:10.1371/journal.pone.0140740)

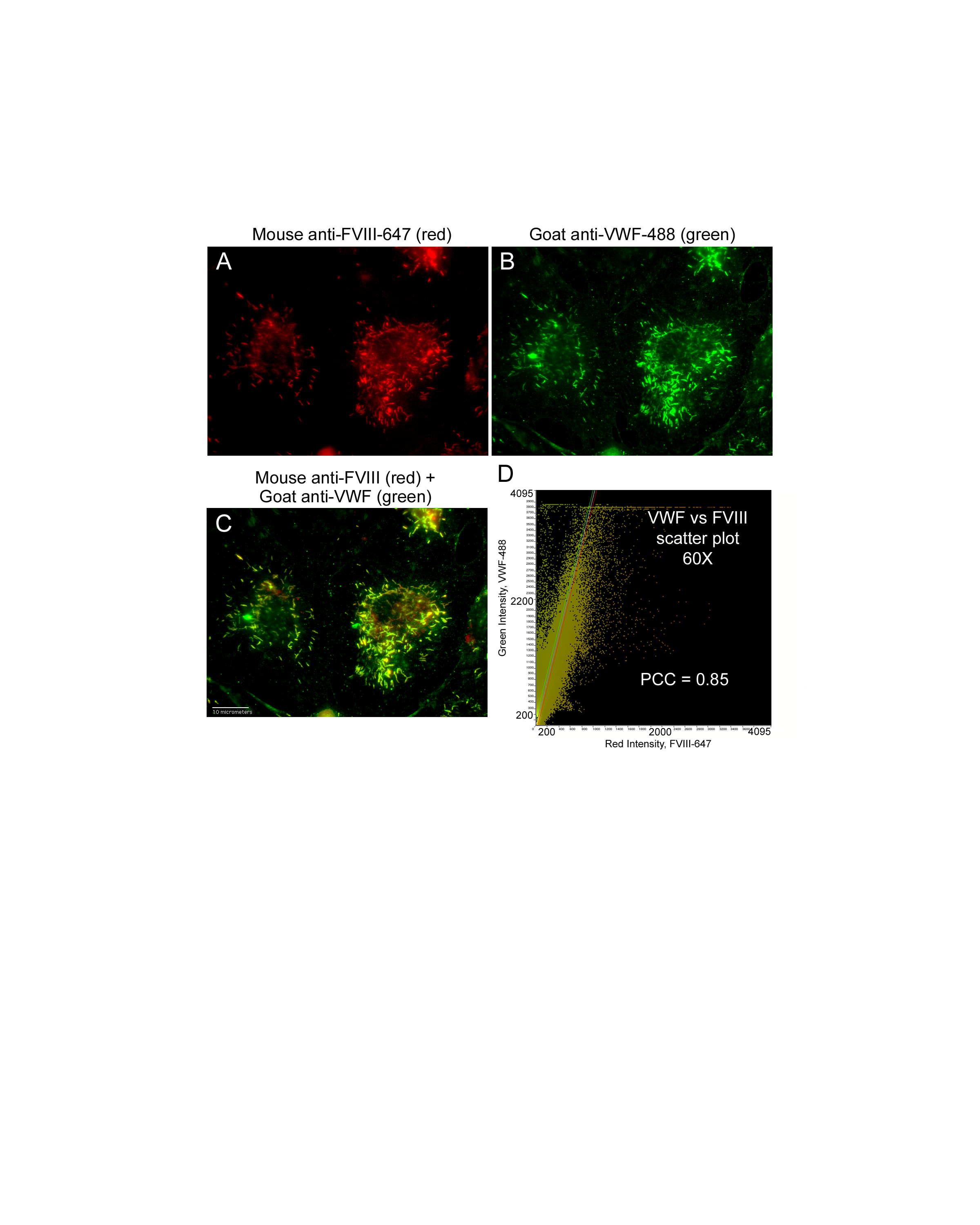

Supplement: S1 Fig — Unstimulated HUVECs were fixed with 1% p-formaldehyde and treated with Triton-X to allow intracellular staining. Cells were stained with mouse monoclonal anti-human FVIII plus chicken anti-mouse IgG AF-647 (red), followed by staining with goat anti-human VWF plus donkey anti-rabbit IgG AF-488 (green). The mouse monoclonal antibody to FVIII is the same one that was used throughout the study. This primary polyclonal goat VWF antibody and both secondary detection antibodies were used only in this set of fluorescent detection experiments. The goat anti-VWF was also used for Western blot detection in Fig 1. The HUVEC images are at 60X: (A) anti-FVIII detection (red); (B) anti-VWF detection (green); and (C) merged image of anti-FVIII plus anti-VWF. Panel D is the intensity scatter plot of the merged image in (C) with the colocalization coefficient (PCC) value that is described later in this article. (TIF) [file pone.0140740.s009.tif]

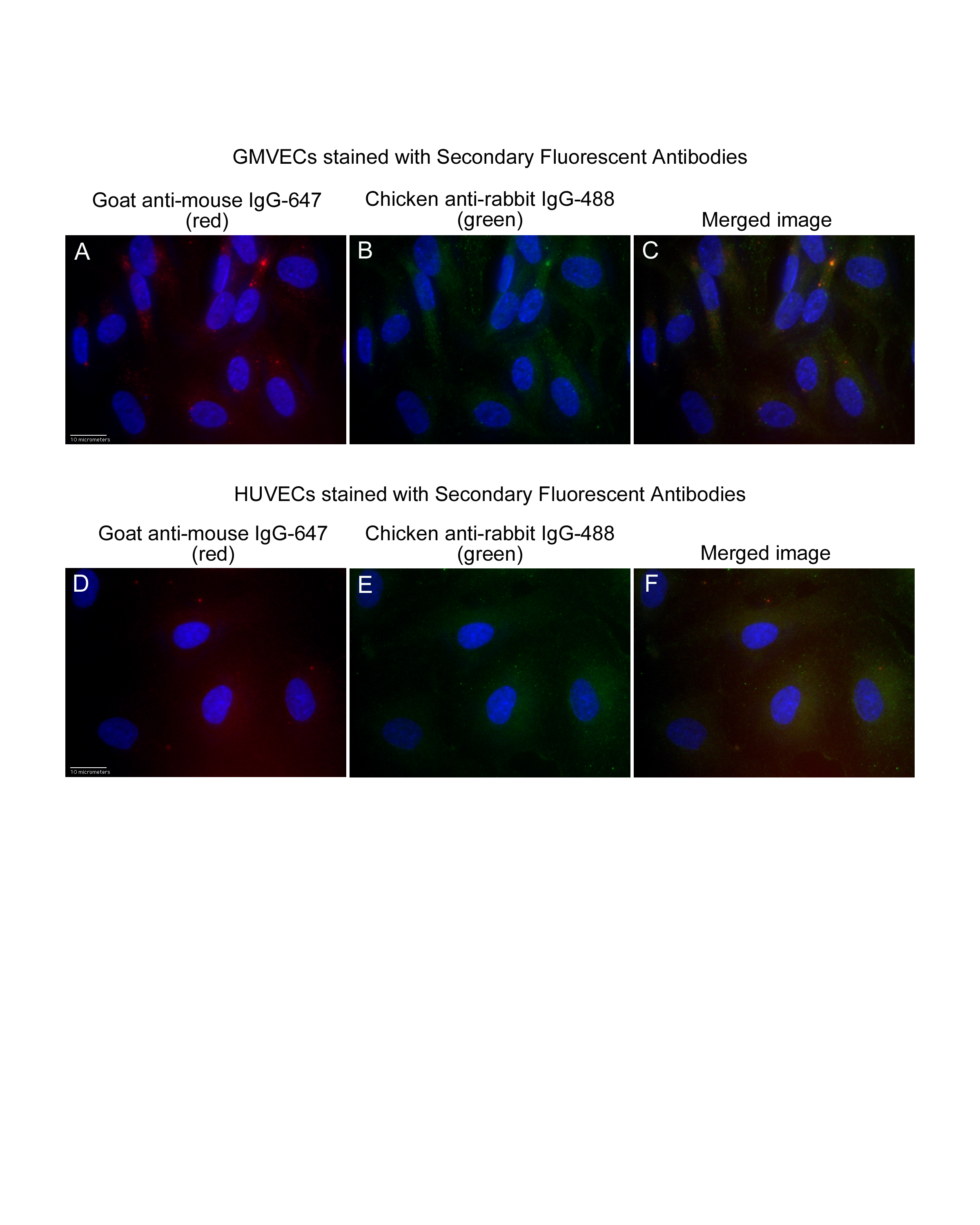

Supplement: S2 Fig — GMVECs (A-C) and HUVECs (D-F) were treated with Triton-X to allow internal staining. Cells were then stained with goat anti-mouse IgG AF-647 (A and D, red) and chicken anti-rabbit IgG AF-488 (B and E, green) secondary detection antibodies at final concentrations of 20 μg/ml before mounting and image acquisition at 60×. Cell nuclei were detected with DAPI (blue). (TIF) [file pone.0140740.s010.tif]

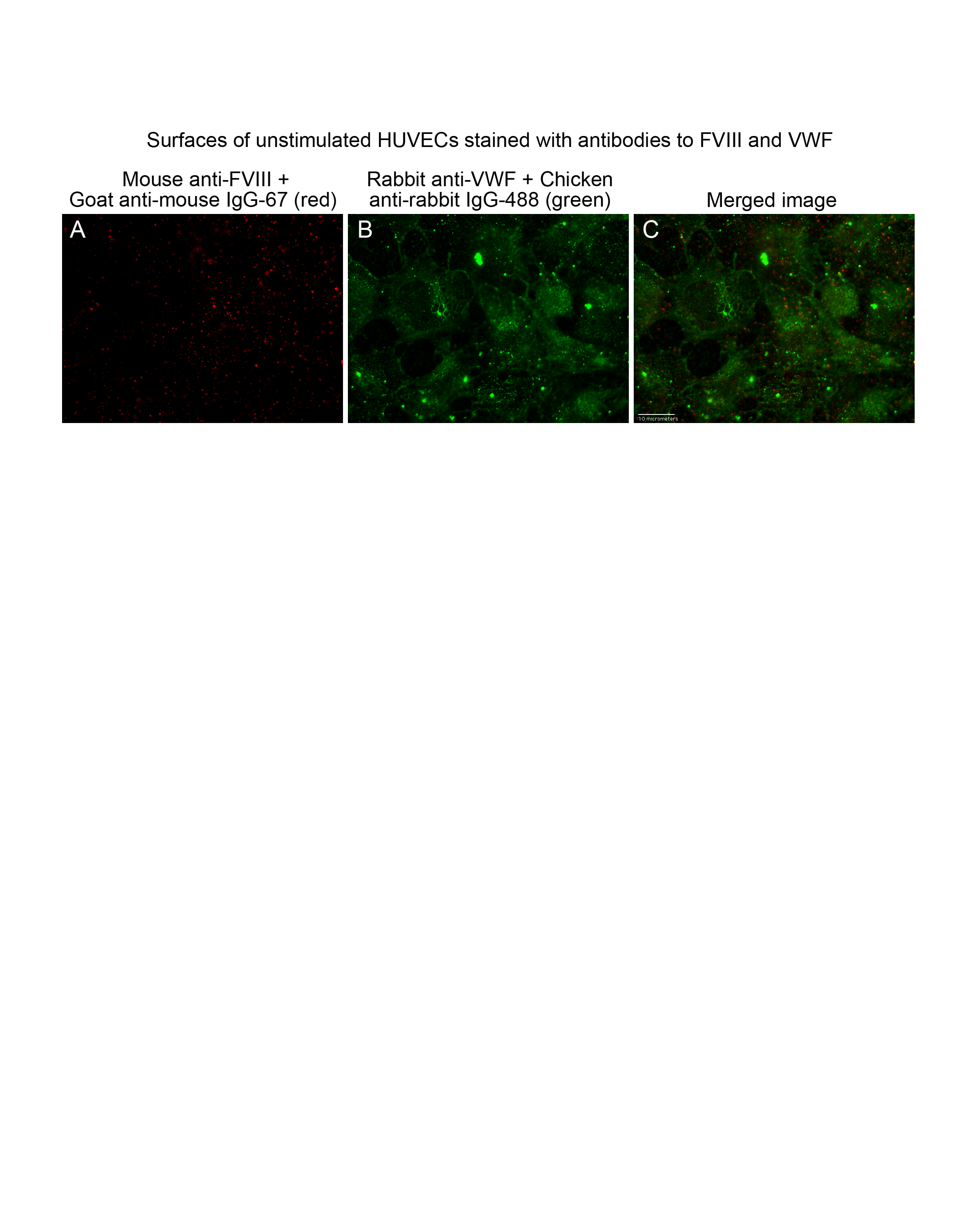

Supplement: S3 Fig — HUVECs were washed and fixed before surfaces were stained with mouse anti-FVIII + goat anti-mouse IgG AF-647 and rabbit anti-VWF + chicken anti-rabbit IgG AF-488. Single channel detection images are shown in (A) mouse anti-FVIII (647, red), and in (B) rabbit anti-VWF (488, green), with the merged image in (C). (TIF) [file pone.0140740.s011.tif]

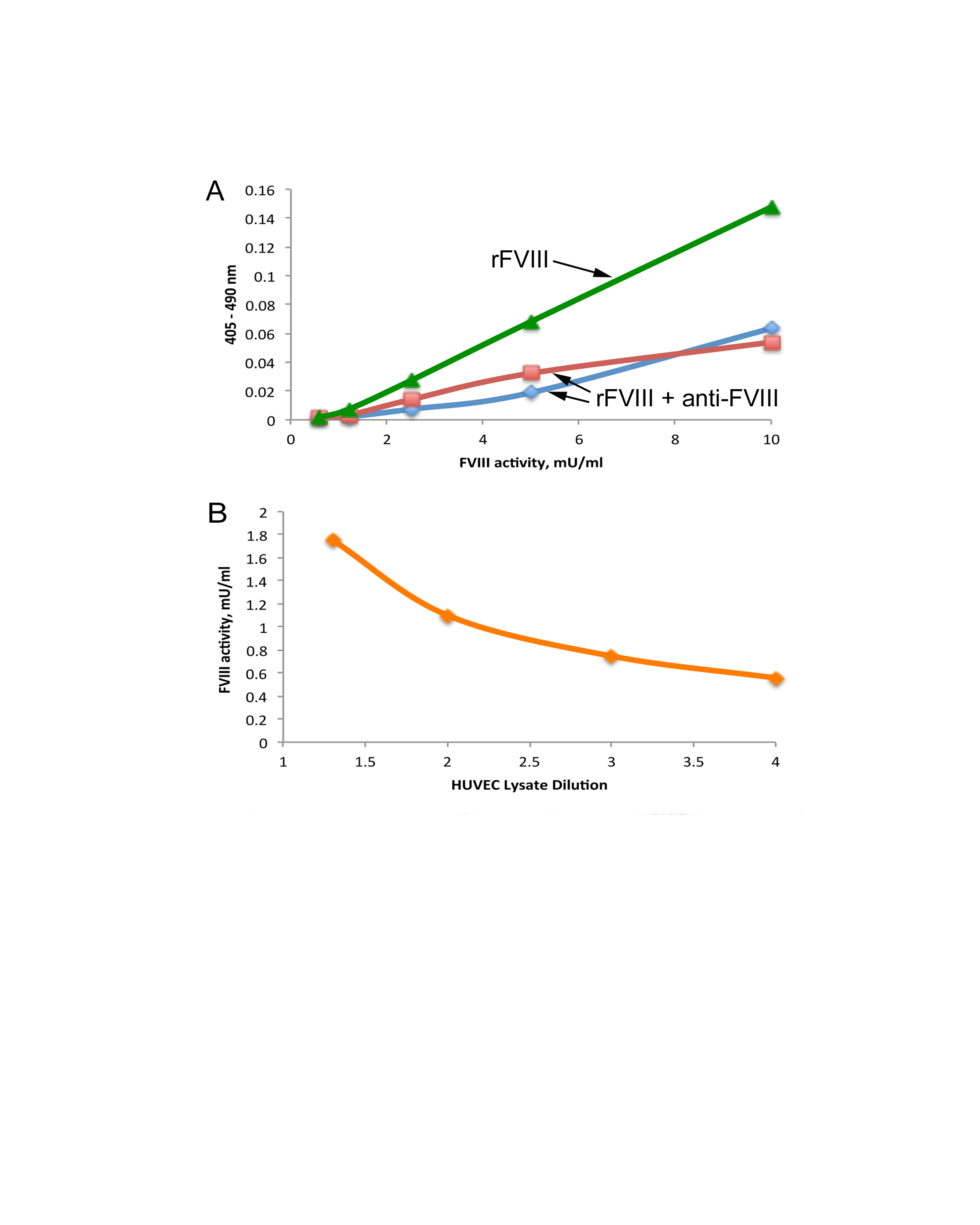

Supplement: S4 Fig — (A) FVIII activities, ranging from 0.6–10 mU/ml, in dilutions of rFVIII, were inhibited by addition of 10 μg/ml mouse anti-human FVIII antibody (clone RFF-VIIIC/8) for 10 min (on ice) prior to the start of the chromogenic Coatest assay. The green triangles represent rFVIII without antibody addition and the red squares and blue diamonds are 2 separate rFVIII dilutions with final concentrations of 10 μg/ml anti-FVIII. (B) FVIII activity was measured in HUVEC cell lysates diluted 1.3-, 2-, 3- and 4-fold in 1% BSA/PBS. (TIF) [file pone.0140740.s012.tif]
