# Supplementary material for: Factor VIII Is Synthesized in Human Endothelial Cells, Packaged in Weibel-Palade Bodies and Secreted Bound to ULVWF Strings
Source: PLoS One. 2015 Oct 16;10(10):e0140740. doi: 10.1371/journal.pone.0140740 (PMC4608722; doi:10.1371/journal.pone.0140740)
Supplement: S1 Table — PCR amplified products were detected using TaqMan Gene Expression Assays with 6-carboxyfluorescein-labeled probes that span target exon junctions (Life Technologies). (PDF) [file pone.0140740.s013.pdf]

S1 Table. TaqMan Gene Expression Assay Probes

Gene	Taqman Probe	Amplicon Length
F8	Hs00252034_m1	127
VWF	Hs01109446_m1	56
AVPR2	Hs00181055_m1	68
GAPDH	Hs02758991_g1	93

PCR amplified products were detected using TaqMan Gene Expression Assays with 6-carboxyfluorescein-labeled probes that span target exon junctions (Life Technologies).
