# Supplementary material for: Factor VIII Is Synthesized in Human Endothelial Cells, Packaged in Weibel-Palade Bodies and Secreted Bound to ULVWF Strings
Source: PLoS One. 2015 Oct 16;10(10):e0140740. doi: 10.1371/journal.pone.0140740 (PMC4608722; doi:10.1371/journal.pone.0140740)
Supplement: S2 Table — The AF-labeled fluorescent secondary antibodies were supplied at 2 mg/ml and were used at a final concentration of 20 μg/ml in 1% BSA/PBS (Life Technologies). (PDF) [file pone.0140740.s014.pdf]

S2 Table. Alexa Fluor (AF)-labeled secondary antibodies

**Alexa Fluor (AF)-labeled secondary antibodies**	Catalogue #
Chicken anti-rabbit IgG AF-488	A21441
Chicken anti-rabbit IgG AF-647	A21443
Goat anti-mouse $F(ab')_2$ fragment-IgG AF-647	A21237
Goat anti-mouse IgM AF-488	A20142
Donkey anti-mouse IgG AF-488	A21202
Chicken anti-goat IgG AF-647	A21469

The AF-labeled fluorescent secondary antibodies were supplied at 2 mg/ml and were used at a final concentration of 20 μg/ml in 1% BSA/PBS (Life Technologies).
