# Supplementary material for: Factor VIII Is Synthesized in Human Endothelial Cells, Packaged in Weibel-Palade Bodies and Secreted Bound to ULVWF Strings
Source: PLoS One. 2015 Oct 16;10(10):e0140740. doi: 10.1371/journal.pone.0140740 (PMC4608722; doi:10.1371/journal.pone.0140740)
Supplement: S3 Table — Average number of PCR cycles ± standard deviation to reach the threshold fluorescent signal detection in real-time PCR using TaqMan probes. Data are from 4–7 separate RNA isolations from each cell type. Threshold cycle numbers are means from 3–7 PCR analyses with triplicate measurements within each PCR experiment. (PDF) [file pone.0140740.s015.pdf]

S3 Table. Threshold cycle numbers for each cell type

	Real-time PCR Threshold Cycle (C_T)			
	GAPDH	*F8*	*VWF*	*AVPR2*
GMVECs	17.37 ± 0.12	33.04 ± 0.14	21.02 ± 0.19	36.85 ± 0.91
HUVECs	16.20 ± 0.11	31.32 ± 0.11	20.60 ± 0.18	37.66 ± 0.66
Fibroblasts	16.95 ± 0.10	30.24 ± 0.09	34.61 ± 0.48	34.88 ± 0.32

Average number of PCR cycles ± standard deviation to reach the threshold fluorescent signal detection in real-time PCR using TaqMan probes. Data are from 4-7 separate RNA isolations from each cell type. Threshold cycle numbers are means from 3-7 PCR analyses with triplicate measurements within each PCR experiment.
