# Supplementary material for: Factor VIII Is Synthesized in Human Endothelial Cells, Packaged in Weibel-Palade Bodies and Secreted Bound to ULVWF Strings
Source: PLoS One. 2015 Oct 16;10(10):e0140740. doi: 10.1371/journal.pone.0140740 (PMC4608722; doi:10.1371/journal.pone.0140740)
Supplement: S4 Table — The average mRNA copy numbers for each cell type at the threshold cycle (CT shown in S3 Table) was normalized to the mean copy number of GAPDH for each cell type at the threshold cycle. The copy number = 2-CT. (PDF) [file pone.0140740.s016.pdf]

S4 Table. Comparison of mRNA copy numbers

	Copy numbers normalized to *GAPDH*		
	F8	*VWF*	*AVPR2*
GMVECs	1.92 (10^{-5})	7.94 (10^{-2})	1.37 (10^{-6})
HUVECs	2.81 (10^{-5})	4.74 (10^{-2})	3.47 (10^{-7})
Fibroblasts	9.98 (10^{-5})	4.83 (10^{-6})	4.01 (10^{-6})

The average mRNA copy numbers for each cell type at the threshold cycle (C_T shown in Table S3) was normalized to the mean copy number of *GAPDH* for each cell type at the threshold cycle. The copy number = 2^{-C_T}
