# Supplementary material for: Factor VIII Is Synthesized in Human Endothelial Cells, Packaged in Weibel-Palade Bodies and Secreted Bound to ULVWF Strings
Source: PLoS One. 2015 Oct 16;10(10):e0140740. doi: 10.1371/journal.pone.0140740 (PMC4608722; doi:10.1371/journal.pone.0140740)
Supplement: S5 Table — Fluorescent intensities were measured in fibroblasts internally stained separately with rabbit anti-VWF plus two different secondary detection antibodies or with these secondary detection antibodies alone. Staining with secondary chicken anti-rabbit IgG-488 resulted in higher fluorescent intensities than rabbit anti-VWF + chicken anti-rabbit IgG-488. (PDF) [file pone.0140740.s017.pdf]

S5 Table. Fluorescent intensities of fibroblasts stained with secondary detection antibodies with and without primary antibodies to VWF

**Rabbit anti-VWF + Chicken anti-rabbit IgG-488**	
Total Intensity	Mean Intensity
1.33 (10^8)	377
**Secondary Chicken anti-rabbit IgG-488**	
Total Intensity	Mean Intensity
2.28 (10^8)	647
**Rabbit anti-VWF + Chicken anti-rabbit IgG-647**	
Total Intensity	Mean Intensity
5.85 (10^7)	166
**Secondary Chicken anti-rabbit IgG-647**	
Total Intensity	Mean Intensity
5.23 (10^7)	149

Fluorescent intensities were measured in fibroblasts internally stained separately with rabbit anti-VWF plus two different secondary detection antibodies or with these secondary detection antibodies alone. Staining with secondary chicken anti-rabbit IgG-488 resulted in higher fluorescent intensities than rabbit anti-VWF + chicken anti-rabbit IgG-488.
